# Aerogels for Phase-Change Materials in Functional and Multifunctional Composites: A Review

**DOI:** 10.3390/ma17174405

**Published:** 2024-09-06

**Authors:** Katarzyna Suchorowiec, Natalia Paprota, Kinga Pielichowska

**Affiliations:** Department of Biomaterials and Composites, Faculty of Materials Science and Ceramics, AGH University of Krakow, Al. Mickiewicza 30, 30-059 Krakow, Poland; suchorowiec@agh.edu.pl (K.S.); npaprota@agh.edu.pl (N.P.)

**Keywords:** phase-change materials, aerogels, functional composites

## Abstract

Phase-change materials (PCMs) have gained more attention during the last few decades. As the main function of these materials is to store and release energy in the form of latent heat during phase transitions, they perfectly fulfill the direction of modern research focused on energy-related topics. Although they have basic energy-related properties, recent research shows a need to upgrade those materials in terms of improving their common drawbacks like shape stability, leakage, and poor conductivity. The research related to PCM-based composites leads to imparting some additional functional properties such as different types of conversion abilities or extra performance such as shape memory and thermal protection. Together with a new emerging material group—aerogels (AGs), extra-light and highly porous matrices—PCMs could become functional and multifunctional materials. AG-PCM composites could be implemented in a large variety of applications in different sectors like energy, buildings, medical, defense, space technologies, and more. This study aims to help summarize current trends, methods, and works on PCM–aerogel composites in terms of developing new functional materials, especially for energy conversion purposes but also for improved conductivity, mechanical properties, and flame retardancy.

## 1. Introduction

The energy demand is continuously increasing with human development. The International Energy Agency predicts that energy demand will increase at an average annual rate of 1.3% every year [1]. As the world is exposed to an energy crisis and governments and international institutions are creating and implementing policies to reduce carbon emissions in particular, there is a need to develop sustainable energy sources and systems that are green, clean, and low-cost.

One sustainable solution is thermal energy storage (TES) technologies. TES technologies are mainly focused on latent heat storage, which can be achieved implementing phase-change materials (PCMs), but they can also perform in sensible heat storage or thermochemical storage fields. The advantage of latent heat storage is ascribed essentially to the large amounts of stored energy compared to the small temperature changes, but there are still some aspects that need to be solved in order to maximize the effectiveness of thermal energy storage of PCMs and their general performance. The main concerns are related to PCMs’ leakage during solid–liquid transitions and their low thermal conductivity. In order to limit the phase-change material leakage, shape stabilization is essential. For this purpose, encapsulation or porous materials are usually used. Li et al. [2] proposed a novel n-octadecane@PMMA/TiO_2_ hybrid shell PCM with excellent thermal performance. Expanded perlite with expanded graphite was used for octadecanol shape stabilization by Lv [3]. The leakage of the obtained PCM was effectively improved. For thermal conductivity improvement, some metallic, carbon, and other additives are often used. Li and co-workers [4] significantly improved paraffin’s thermal conductivity by adding modified carbon nanotubes (CNTs), while metal foam was used by Yu [5] to enhance paraffin’s low thermal conductivity. However, solving those problems would not be enough for the future world outlook, so new types of emerging material aerogels may be useful.

Aerogels (AGs) are a group of three-dimensional materials with a very high porosity and a low density. Thanks to their macroscopic morphology, they have been successfully implemented in fields like thermal insulation [6], space technologies [7], energy storage [8], sensors [9], etc. Their unique microstructure also makes them a perfect solution to overcome the drawbacks of PCMs in terms of leakage and shape stabilization. Combining PCMs with AGs is a new way to develop sustainable materials in energy-related fields, as AG’s porous microstructure can absorb large amounts of PCM and prevent leakage because of strong capillary forces and surface tension. The latent heat of composites remains, respectively, high compared to pure PCMs [10].

Some works focus on different ways of preparation or classification of AG-PCM composites [11] or their applications [10]; however, the directions of current research not only focus on the energy storage, shape stabilization, or leakage prevention of AG-PCM composites but are heading more towards creating new functional and multifunctional materials that can be capable of smart performance conversions or possess additional unique properties like flame retardancy, enhanced flexibility, shape memory, and adjusted thermal conductivity.

While numerous studies have explored the individual components of AGs [12,13,14,15] and PCMs [16,17,18,19,20], there remains a significant gap in the literature on their combined use in functional and multifunctional composites. Taking into account current trends, this work focuses on a brief but comprehensive review of the achievements of the last ~5 years in the field of AG-PCM composites, paying attention mainly to the functionality and not only the thermal energy storage performance of these materials. This review aims to present material design, synthesis methods, properties, applications, and future perspectives of AG-PCM composites. This review is crucial as it consolidates the current state of research, identifies key challenges, and highlights innovative strategies for improving the performance of AG-PCM composites.

In this review, different types of AG-PCM functionalities are discussed. The first part focuses on the conversion-based multifunctionalities of AG-PCM composites, while the second part emphasizes additional properties for functional composites, followed by a brief discussion and outlook on the field of PCMs combined with AGs and their future applications.

## 2. Phase-Change Materials

**Figure 1:**
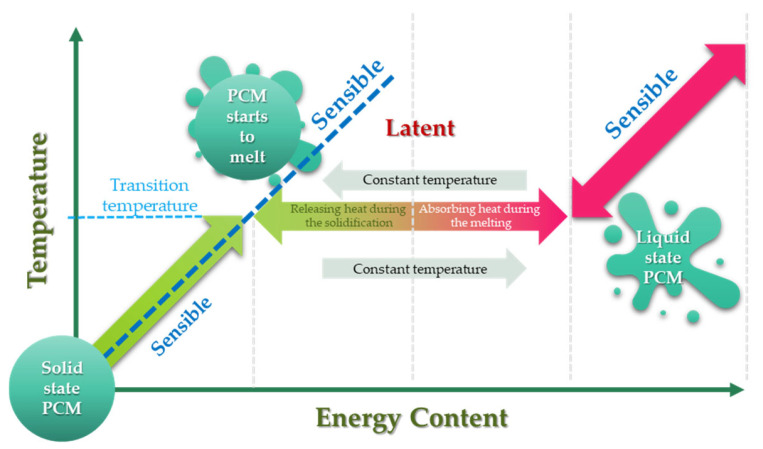
Temperature and stored energy in the SHS and LHS systems. Modified from [22] with permission from Elsevier.

**Figure 2:**
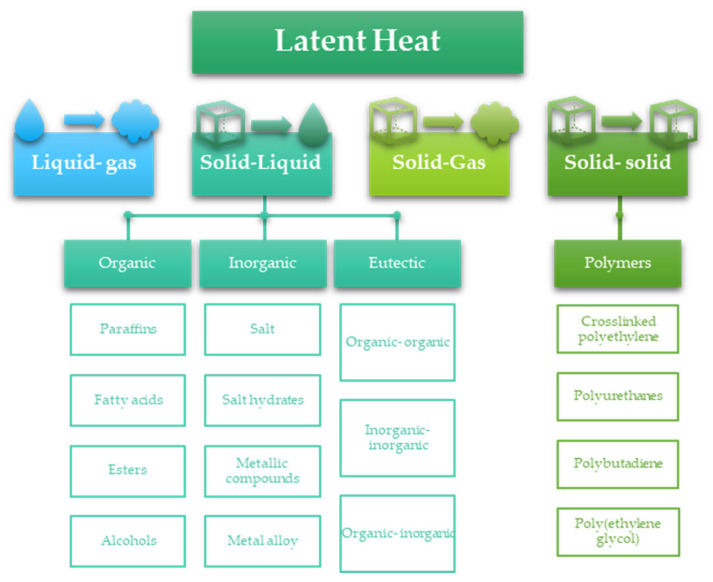
Classification of PCMs.

PCMs are a group of materials that can store energy in the form of latent heat during ongoing phase transitions in defined temperature ranges (Figure 1). PCMs can be classified within several classifications: type of phase transitions (e.g., solid–liquid, solid–solid, liquid–gas, etc.), type of material (organic, inorganic, eutectic), phase-change temperature range (low temperature, high temperature) [21]. The basic classification of PCMs is shown in Figure 2.

The most widely used PCMs are organic PCMs, mainly paraffins, fatty acids, and alcohols or polyglycols. Among them, paraffins play a crucial role as PCMs; they are mixtures of alkanes with a carbon number in the range from twenty to forty. The amount of heat that they can store depends mainly on the molar mass [23,24]. The main advantages of using organic PCMs are good thermal stability and reliability, small supercooling, narrow phase-transition temperature range, safety for humans, and neutrality for the environment.

Among inorganic PCMs, four main groups can be distinguished: salt, salt hydrates, metallic compounds, and metal alloys. The most popular group consists of salts and salt hydrates containing crystallized water. The main difference from organic materials is that they exhibit a higher melting temperature and fusion heat [25]. Inorganic PCMs, especially inorganic salts like eutectic mixtures of CaCl_2_ and LiC [26] or LiCl–LiOH [27], can be used as thermal energy storage materials that could operate in high temperatures. Compared to low molecular weight organic PCMs, inorganic materials may cause corrosion or suffer from a higher supercooling effect, which is an effect of poor nucleating ability [28].

The solid–solid phase change mainly occurs in polymeric materials where the phase change is ascribed mainly to the conformational changes that occur within the polymeric chains, and the material remains in a solid state, e.g., changing states from a crystalline to an amorphous state. The main advantages of these processes are a small volume change and no leakage during the phase transition. What is more, polymeric PCMs have the advantage of further modification to meet the industry and end-product requirements, but at the same time, they may also suffer from supercooling.

As PCMs’ main implementation purposes are energy-related applications, it is crucial to mention that in terms of energy harvesting systems and solutions, PCMs will require some additional modifications because of their low thermal conductivity and flammability. In particular, to create functional or multifunctional materials, they need other supporting and functionality-providing materials.

## 3. Shape Stabilization

The application of phase-change materials is often limited by their leakage during the solid–liquid phase transition; therefore, the shape stabilization of PCMs is necessary. Many different approaches have been tested for this purpose, but the greatest interest is in the use of encapsulation and porous materials [29,30,31].

### 3.1. Microencapsulation

**Figure 3:**
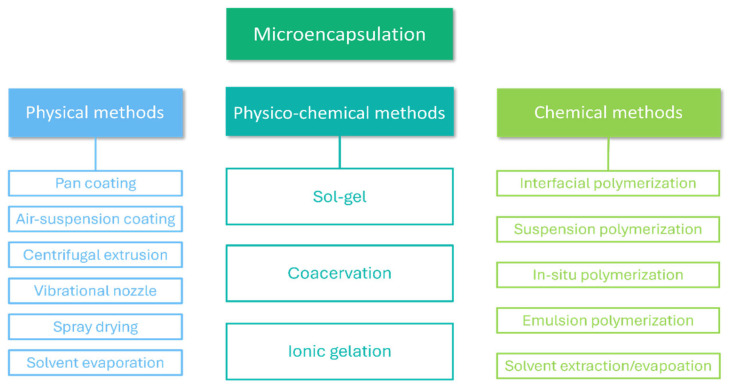
Different techniques of PCM microencapsulation.

Microencapsulation is a technique in which one material is covered by another. Microencapsulated phase-change materials consist of two main components: a PCM core and a polymer or inorganic shell that serves as the PCM container. Microcapsules can have both regular and irregular shapes. The main advantages of PCM encapsulation include controlling PCM volume changes during phase transition; isolating PCM from the environment, which acts as a protective barrier and significantly limits degradation; increasing surface area, which improves thermal conductivity; reducing corrosion; and increasing the compatibility of PCMs with other materials [29,31,32]. Different techniques for PCM microencapsulation are available—Figure 3.

### 3.2. Porous Materials

**Figure 4:**
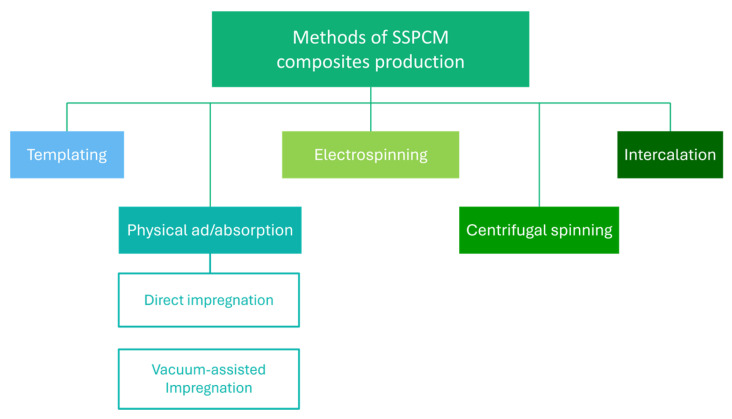
Methods to produce SSPCM.

Different types of porous materials are great for the shape stabilization of phase-change materials (SSPCM). These composites are formed by embedding PCMs into a supporting material. Over the past decade, they have garnered significant attention because of their remarkable capability to maintain their shape through numerous thermal cycles without the need for encapsulation. Porous supports are classified according to pore size into four types: macroporous (greater than 50 nm), mesoporous (2–50 nm), microporous (less than 2 nm), and hierarchical porous materials, which range from macroporous to microporous. Among these porous supports are the following: metallic (copper or aluminum foam), carbon-based (expanded graphite, CNTs), polymers (polyethylene, polyurethane foams), clay minerals (vermiculite, expanded perlite), and mesoporous silica [29,31,33,34]. For example, Qui et al. [35] used a biometric AlN ceramic with a unique hierarchical pore structure for PEG shape stabilization. As a result, the leakage of the phase-change material was limited in the obtained composites. At the same time, the addition of alumina (Al_2_O_3_) nanoparticles significantly improved the PEG’s thermal conductivity. Expanded graphite was also used for PEG shape stabilization [36]. Its advantage is not only its porous microstructure but also its energy conversion capacity. The obtained EG/PEG composites were characterized by a high latent heat of phase change and thermal conductivity with limited supercooling and leakage of PEG. SSPCM composites are possible to obtain using different methods—Figure 4.

## 4. Aerogels

AGs are a group of materials with outstanding porosity and a large number of nanopores. They are ultra-light with a well-developed 3D network structure. These materials have gained a lot of attention in recent years because of their unique properties coming from a low density together with ultrahigh porosity: high specific surface area and mass transfer. Their unique properties have also found wide usage in a variety of applications such as energy storage materials, catalysts, catalytic supports, chemical adsorbents, thermal insulators, and sound insulators [13].

AGs differ from other porous materials by the following:

- Porosity—AGs are characterized by very high porosity, typically ranging from 90% to 99.8%; other porous materials can also be highly porous but do not reach the same extreme levels as AGs.
- Pore size—pore size in AGs is typically nanosized, and this also influences their unique properties.
- Structure—AGs have a highly interconnected network structure, often derived from a gel in which the liquid component has been replaced with gas without causing significant collapse of the gel structure. This network of interconnected nanostructures is responsible for the low density and high surface area of the material.
- Density—AGs are among the lightest solid materials known, with densities as low as 0.001 g/cm^3^. This extremely low density is a direct consequence of their high porosity and nanoporous structure. Other porous materials can also be lightweight, but they typically have higher densities than AGs because of their less extreme porosity.

**Figure 5:**
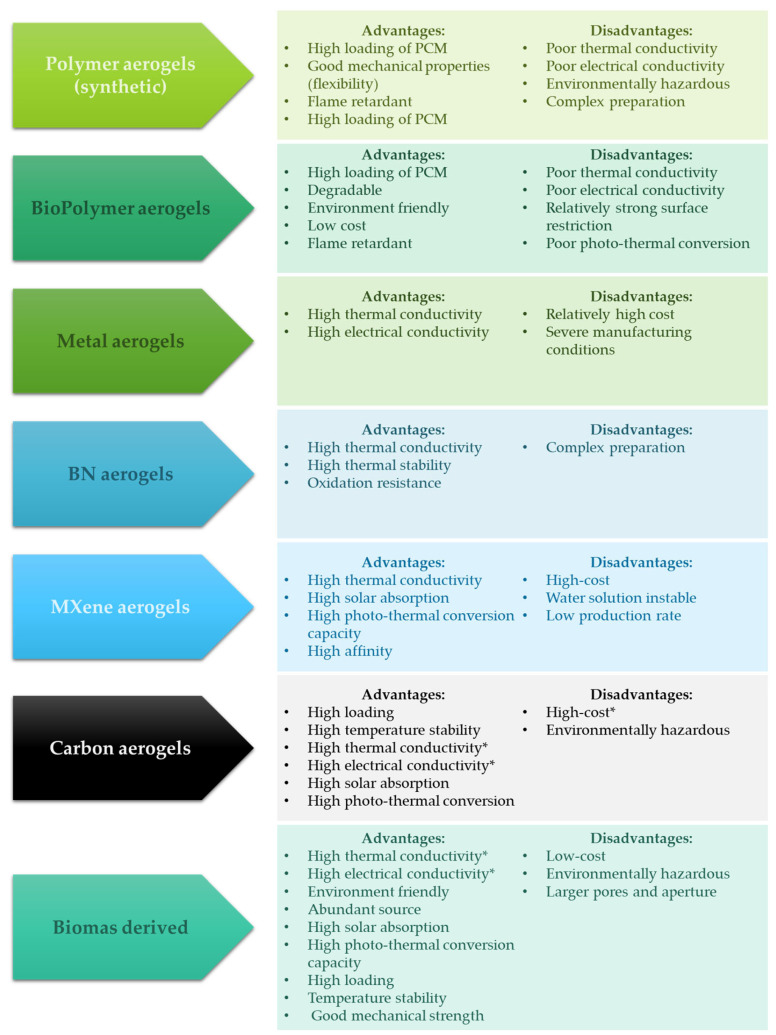
Advantages and disadvantages of different kinds of AGs (* depends on the type of carbon material).

Two main classifications of AGs can be distinguished by the material and the method of preparation. Taking the material classification into consideration, oxides, carbon, polymeric, metallic, biomass-delivered, and non-oxide ceramic AGs can be identified. A summary of the advantages and disadvantages of AG types is presented in Figure 5.

The fabrication classification mainly focuses on a sol–gel method that includes transforming some precursors into organic or inorganic gel with a high crosslinking ratio. The second step of the sol–gel method is to exchange the liquid solvents with gas. Usually, different methods are implemented to obtain the following: lyophilization, supercritical drying, and ambient pressure drying. When the liquid is replaced by gas, a 3D framework could emerge. Metal AGs are mainly prepared via traditional methods such as sol–gel, dealloying, templating, and powder metallurgy [10].

Different approaches are used for carbon AGs. The most commonly used technique is the carbonization of an organic precursor (organic AG, obtained by polymerization or biomass pretreatment and then dried). However, some well-structured material-delivered AGs like graphene, graphene oxide (GO), and CNTs require the implementation of the sol–gel method where there is no need for carbonization [37]. The sol-gel process represents a straightforward method for synthesizing graphene aerogels.

The sol-gel method involves the formation of sol containing precursors of the desired material, which are dispersed in water or other solvent to form a stable sol. Then, the chemical reactions within the sol lead to the formation of a network-like self-assembly of nanosheets or nanotubes, facilitated by hydrogen bonding, van der Waals forces, or covalent crosslinking agents. Over time, the sol undergoes gelation, forming a three-dimensional porous network. The last steps involve an evaporation or freeze-drying process to eliminate the solvent. As an example, for graphene AGs, the process begins with aqueous GO. During the sol-gel transition, GO is reduced using chemical reducing agents or hydrothermal treatment, resulting in a reduced graphene oxide (rGO) sol. The rGO sol then undergoes gelation through self-assembly, forming a stable three-dimensional hydrogel. To obtain reduced graphene oxide aerogels (referred to as graphene aerogels, GAs), the rGO hydrogels are subjected to freeze-drying [38].

The main difference between graphene and graphene AGs is that graphene itself is a 2D material and cannot provide proper shape stabilization for PCMs, in contrast to carbon aerogels, which are 3D materials with excellent porosity which is appropriate to close the PCM within their structure. Also, there could be a difference in properties such as electrical conductivity. When it comes to graphene AGs, they are a three-dimensional network of graphene sheets. Therefore, they combine the properties of AGs with those of graphene, making them even more efficient in terms of energy conversion properties in PCM-based composites. There are also differences in material preparation. Graphene AGs are usually prepared by reducing GO in a solution, forming a gel, which is then dried (usually via freeze-drying or supercritical drying) to create the aerogel, while carbon AGs are usually made in the carbonization process. In terms of future application, carbon aerogels may be more sustainable (because they can be produced via waste carbonization) and more economically available.

The development of 3D printing techniques allowed researchers to obtain a well-designed AG with fixed porosity and pore sizes. These techniques are also widely used for carbon additives, such as ink substrate materials including graphene, GO, and carbon nitride, but also other additives like MXenes, metal nanoparticles, resorcinol formaldehyde, or cellulose [39].

## 5. AG-PCM Functional Composites for Energy Conversion

As mentioned above, PCMs suffer from numerous drawbacks, not only in terms of leakage and shape stability. Pure PCMs themselves exhibit a limited range of applications; however, combining them properly with other materials could lead to the creation of multifunctional composites. Nevertheless, some modifications (nanoparticle doping, fiber-based composites, etc.) that could provide functionality or multifunctionality to the composites are not the ultimate solution because of phase-change-related problems like leakage.

**Figure 6:**
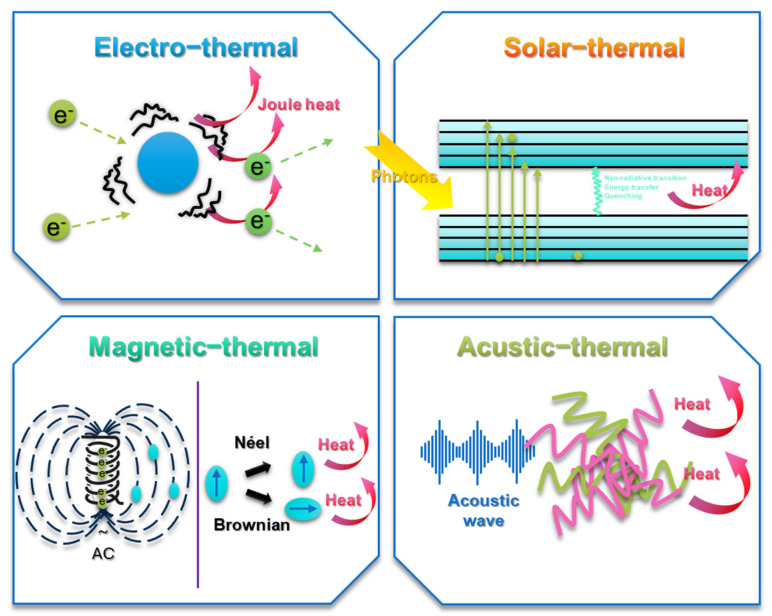
Schematic representation of conversion mechanisms: electro-thermal, solar-thermal, magnetic-thermal, acoustic-thermal.

The most common approach toward PCMs is creating a multifunctional composite with different kinds of conversion abilities like electro-thermal, light/solar–thermal, magnetic–thermal, or acoustic–thermal (Figure 6). Among all the conversion types, electro-thermal conversion and especially light/solar–thermal conversion are widely studied in AG-PCM composites. The remaining two types of conversions, magnetic–thermal and acoustic–thermal, have gained less attention, but there are still some works emerging that cover these topics. In addition, the focus in recent years has been on providing a multi-conversion composite system, especially for solar energy harvesting systems and thermal storage applications.

A comprehensive overview of the properties of AG-PCM for conversion applications is shown in Table 1.

### 5.1. Electric–Thermal Conversion

Electric energy is one of the most popular forms of energy currently used on the planet. This is because it can be produced by power plants, solar panels, and wind turbines, and transmitted through power lines to supply electricity to different locations. This type of energy can be easily converted and has a large flexibility in transmission [68]. On the other hand, thermal energy produced by fossil fuel combustion, nuclear reactions, and photo-thermal collectors cannot be transmitted over long distances. That is why conversion from electricity to heat may be useful in areas such as building temperature control, medical therapy, clothing temperature regulation, and heating [68].

Apart from metallic PCMs, which are not so common, there are no pure PCMs that exhibit the desired electrical properties (the electrical conductivity of pure PCMs ranges from 10^−12^ to 10^−7^ S [69]), which is why these materials require some modifications to meet those properties. Common modifications involve doping with metal fibers, carbon additives, or different types of nanoparticles. Introducing a 3D-structured matrix with outstanding electrical conductivity could not only lead to obtaining a composite with good electrical properties but also provide PCM encapsulation and thus lead to an electro-thermal conversion effect. The main mechanism behind the process is discussed in this section [70].

The mechanism behind the electro-thermal conversion process is mainly based on electrical conductivity and thermal conduction. All kinds of material heat can be transferred by three main different mechanisms: heat conduction, heat convection, and heat radiation. Heat conduction takes place inside a solid or on its surface when contact with a different solid or liquid occurs. This type of heat transfer mechanism is most common in PCMs [71]. In comparison, heat convection occurs only in liquid or gaseous substances, and thermal radiation occurs for all materials above absolute zero temperature. Additionally, PCM heat transfer can be divided into two main groups, electron heat transfer or phonon heat transfer, which are connected with lattice vibration waves, free electrons, and electromagnetic radiation [72].

As the electro-thermal conversion effect is discussed in this section, electrical conductivity should also be discussed. The electrical conductivity of a material depends mainly on the type of material, which has different conducting mechanisms. In metals, free electrons are moved by the potential difference, forming an electric current. In inorganic materials like carbon materials (graphite, graphene, CNTs), the electric current is generated through the potential difference when a single electron can move in between the π- bonds that are created by the single electrons in each of the parallel p orbitals of sp^2^ carbon layers [73]. The last mechanism is present in semiconductor materials and is based on the valence electron–hole mechanism. The electron is transported to the valence band, filling the electron holes and forming an electric current.

The electro-thermal conversion occurs in PCM composites when the current passing through the conductive material creates Joule heat. The heat is absorbed by the PCM and stored in the form of latent heat. To obtain high electro-thermal conversion efficiency, high electric conductivity and high thermal conductivity are required [74]. The electric-to-thermal energy conversion efficiency $\left(\eta_{e}\right)$ can be calculated by following Equation (1) [75]:

(1) $$\eta_{e}=\frac{m∆H}{UIt}$$

m—weight of the sample; ∆H—enthalpy of phase change; U—voltage; I—current; t—time of the conversion process.

**Figure 7:**
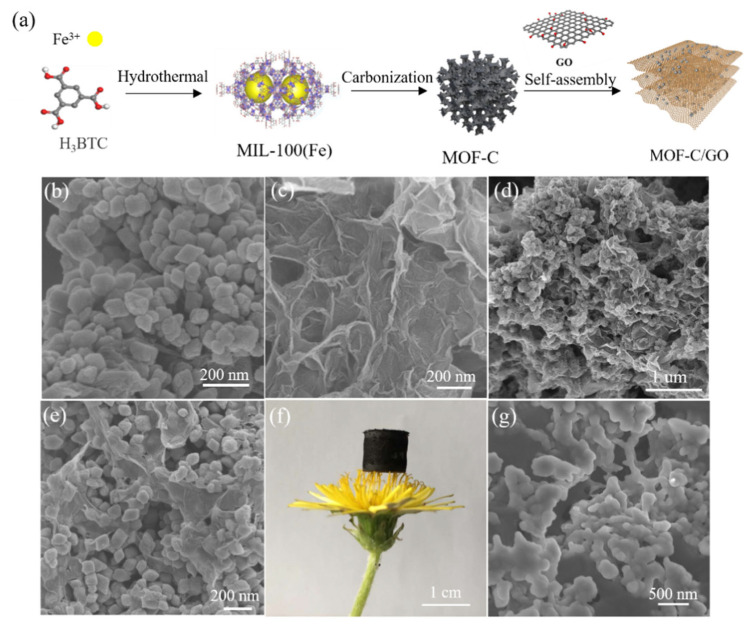
Scheme of sample preparation (**a**). SEM images of MOF-C (**b**), GO (**c**) and MOF-C/GO (**d**,**e**). A photograph of hybrid AG monolith (**f**). SEM image of LA@MOF-C/GO (**g**). Reprinted with permission from Elsevier [40].

In the case of an AG-PCM functional composite with the ability to convert electricity into heat, the material should be projected in a way that overcomes the insulating properties of PCMs. That is why properly designed composites with high-electrical-conductivity AGs not only make the electro-thermal conversion possible but also provide shape stability. Wang et al. [40] designed a PCM composite with a carbon hybrid AG for the temperature management of Li-ion batteries. The hybrid carbon AG was prepared by a hydrothermal method from a metal–organic frame with carbon (MOF-C) and GO dispersion and then impregnated with lauric aid (LA) (Figure 7a). The preparation method provides an AG with a dense network that improves the heat storage and conversion abilities of a composite. Due to the synergistic effect between MOF-C and GO, the prepared material was able to reach a latent heat of 140 J/g, and the denser network also promoted heat/electron transfer and provided a thermal conductivity of the composite of 1.36 W/m⋅K. The conversion efficiency at a low input voltage (2.2 V) reached 90%.

Reduced GO can also work as an efficient additive to provide electrical properties to PCMs. Su et al. [41] presented a novel AG-based PCM composite using rGO cellulose sodium AG and a eutectic mixture of LA with myristic acid (MA) together with SEBS (as polymer crosslinking networks). AGs were prepared through self-gelation and crosslinking of GO with sodium carboxymethyl cellulose colloidal dispersions and freeze-drying followed by a hydrothermal reaction (4 h, 80 °C) to obtain sodium cellulose AGs. The rGO was used because of its better impact on the tensile properties and electrical and thermal conductivity of treaded carboxyl methylcellulose than GO. The composites exhibited high absorption due to the capillary force and pore size and distribution, together with the potential for hydrogen bond formation and π-π interaction. The efficiency of electro-thermal conversion with low electric potential reached 82.3% (29.9% for GO), and good shape stability and almost constant melting and freezing enthalpies were achieved, even after 100 thermal tests.

Deng et al. [42] prepared an AG based on other graphene derivatives as enhancers, using melamine foam (MF) and depositing graphene nanoplatelets (GNTs) and CNTs onto it, followed by a carbonization process and vacuum impregnation with n-octadecane. The samples reached 50 °C in 20 s under a voltage of 6 V, indicating quick energy conversion, which is a result of the presence of GNTs and CNTs within the matrix. The melted octadecane further increases the electrical conductivity because Joule heating takes place and warms the sample, and melting octadecane causes an increase in volume, prompting the CNTs and GNPs within the melamine foam’s pores to slightly shift, forming a web with the ability to conduct electrons. Zhao et al. [43] proposed laminated fabric composed of AG from cellulose nanofiber (CNF) with CNTs impregnated with paraffin wax (PW), and additionally decorated the upper layer with MXene. Placing the material charged by 3 V under the container with ice and water, the ice completely melted within 775 s, and the temperature of the bottom water rose from 1.6 °C to 29.1 °C. This could be ascribed to the synergic enhancing effect of combining highly conductive MXene and CNTs, also indicating the application of the composite for keep-warm clothing in military or high-altitude sports.

Other work has reported carboxymethylcellulose (CMC)-CNT AGs and the vapor deposition of polypyrrole (PPy) together with impregnation with PW, presented by Tao et al. [44]. PPy deposition resulted in higher thermal conductivity (~25% higher than a composite without PPy) and conversion efficiency reaching ~90%, together with an enthalpy of 149.9 J/g at 80.9% of the load of PW. This was the result of an additional layer of PPy that built the thermal transfer channels and improved the heat transport between CNT by coordinating carbon-atom vibration. He et al. [45] reported PW impregnated into a Ag nanoparticle (AgNP)-coated graphene nanosheet AG prepared through hydrothermal reduction and a thermal annealing process. The 3D porous structure of the obtained AG was connected through graphene nanosheets, and efficient heat transfer pathways together with electro- and solar–thermal abilities were achieved through the dispersion of AgNPs. AgNPs changed the pore structure of the base GO AG, enhanced PW adsorption, and achieved a thermal conductivity of 39.35% compared to pure PW. The electro-thermal conversion reached 87.12%, and the temperature reached 85.9 °C when energized at 2.2 V for 143 s with a PW loading of 98.34%. What is more, the composite was also able to generate electricity through the thermoelectric Seebeck effect. Lin et al. [46] proposed a more sustainable way to produce AG-PCM composites with electro-thermal properties using carbonized plant straw and poly(ethylene glycol) 4000 (PEG 4000). The samples obtained from AG carbonized at 1000 °C exhibited high porosity and a structure that led to a composite that exhibited a complete phase change under a voltage of 4 V in 40 s. The temperature reached through electro-thermal conversion was more than 110 °C. The composite had high enthalpy (>185 J/g) and evidence of great shape stability, together with enhanced thermal conductivity with a PEG loading of ~97%.

Zhou et al. [47] presented a different approach to AG-PCM composites, introducing a solid–solid PCM in the form of a polyurethane (PU) and halloysite nanotube (HNT)–graphene AG. The composite was fabricated through air-drying of hydrogel to obtain the HNT–graphene AG and then vacuum-impregnated with PU. The chemical and physical interaction of the PCM with the AG improved the nucleation sites. This resulted in higher latent heat (103.3 J/g) compared to pure PU (86.8 J/g) and an electro-thermal conversion value of 66.3% for samples with 1.17 wt% of HNT–graphene AG.

The works that have been presented indicate the wide range of options to achieve electro-thermal conversion capability in AG-PCMs. However, more research is needed to improve the output and long energy release of the materials to provide a stable source of heat for a large number of working cycles.

### 5.2. Solar–Thermal Conversion

The worldwide energy demand is continuously increasing. Problems caused by fossil fuels have turned researchers’ attention onto renewable energy sources like wind, sun, etc., that are freely available and have a positive environmental impact. The sun is basically an inexhaustible source of energy but also has limitations like unavailability at night or on cloudy days [76]. Therefore, research should focus on an efficient way to store and release this energy when it is needed. PCMs that can store energy in the form of latent heat could help solar–thermal equipment to store as well as capture more energy [77].

The light-to-heat conversion can occur following one of three different mechanisms. The mechanism behind the conversion depends mainly on the type of material that has the ability to convert light into heat [78]. For metals, plasmonic heating is responsible for this effect; it is also known as surface plasmon resonance. The conversion following this mechanism takes place when a plasmonic metal is exposed to incident light. The electrons at the surface of the metal are excited by photons of light, and they move parallel to the surface. The surrounding medium is heated when the [79].

In semiconductor materials, the light–thermal conversion mechanism is driven by electron–hole generation and relaxation mechanisms. When the semiconductor is exposed to sunlight irradiation with energy higher than the bandgap, the hole is generated by the valence electrons breaking the bond with the parent atom [80]. When the relaxation of electron–hole pairs to the bond edge occurs, heat is generated and transferred to the surrounding medium. In these kinds of materials, the conversion depends mainly on the bandgap width. If the bandgap is narrower, there is a higher possibility of electron–hole generation; the opposite occurs for larger bandgaps.

The most common materials used for AGs for support and function in AG-PCM composites are polymers and carbon materials. The main mechanism of solar–thermal conversion, especially in carbon materials, is related to the thermal vibrations of molecules when incident light is absorbed at different wavelengths by the closely spaced energy levels of loosely held π electrons, and heat is generated during the relaxation of the excited electrons to their ground state [44]. This works especially for carbon materials (graphene, GO, CNTs, etc.) due to the closely spaced energy levels of the loosely held π electrons; thus, these materials possess a good ability to absorb sunlight [81].

The solar-to-thermal energy conversion efficiency $\left(\eta_{s}\right)$ can be calculated by following Equation (2) [48]:

(2) $$\eta_{s}=\frac{m∆H}{PS(T_{c}-T_{s})}$$

m—weight of the sample; ∆H—enthalpy of phase change; P—irradiation intensity; $T_{s}\;$ and $T_{c}$—start and end times of the phase-change process.

In terms of solar–thermal conversion, organic PCMs may suffer from inherent inferior light absorbance, poor thermal conduction, and weak shape stability, which may cause serious restrictions in the absorption, conversion, and utilization of solar energy [57].

**Figure 8:**
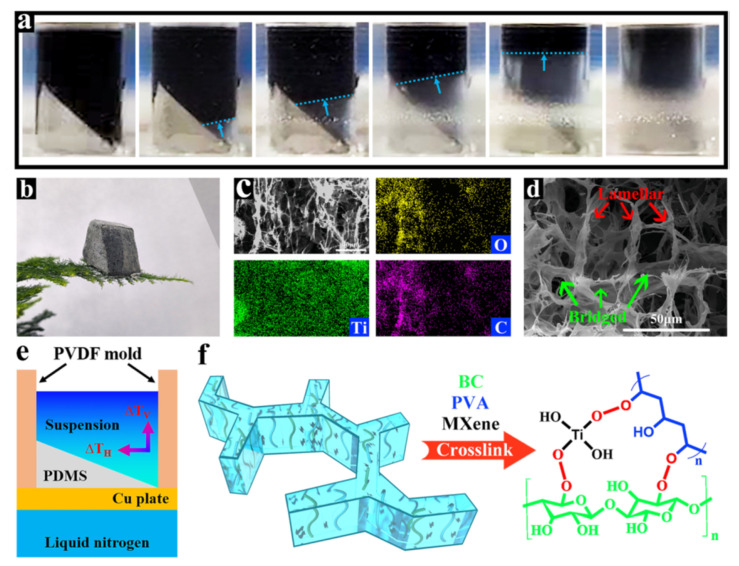
(**a**) Photographs of the bidirectional freeze-casting process for mixed solutions. (**b**) Photograph of BPM AG. (**c**) EDS mapping images of BPM AG for C, O, and Ti. (**d**) SEM of the detailed structure of the BPM AG. (**e**) Schematic diagram of bidirectional freeze-casting mechanism. (**f**) The influence mechanism of the as-synthesized BPM AGs. Reprinted with permission from Elsevier [51].

Luo et al. [48] prepared an AG-PCM composite based on PVA-CNT AG and PEG 6000. The material not only exhibits an excellent light–thermal conversion efficiency of 89.6%, mainly ascribed to the addition of CNTs as they can increase the efficiency of phonon capture, but also has an improved thermal conductivity (0.568 W/m⋅K), which is crucial for this type of functional materials and is 157% higher than pure PEG 6000, and therefore can efficiently control the temperature. In work presented by Ai et al. [49], a Ag-PCM functional composite was prepared for the first time via the facile exfoliation of montmorillonite, the ecofriendly self-assembly and freeze-drying of montmorillonite nanosheets (MNs) and PVA with the addition of rGO for thermal conductivity enhancement and sunlight absorbability. The composite showed an ultrahigh encapsulation ratio of 98.5 wt%, and a high phase-change enthalpy of 191.2 J/g together with effectively enhanced thermal conductivity from 0.300 W/m⋅K to 0.418 W/m⋅K. Solar–thermal energy conversion efficiency reached 91.85% due to high sunlight absorption capacity due to the black appearance of the composite, the efficient photon–electron–phonon excitation of the π bond in rGO, and the exceptional thermal conductivity achieved through the constructed 3D conductive network, which were most likely responsible for the excellent solar–thermal conversion performance. Other work by Li et al. [50] presented a multilayered PVA AG reinforced by boron nitride nanosheets (BNNs) coated with polydopamine (PDA)-modified tetrapod zinc oxide (T-ZnO) hybridized particles and impregnated with PEG 8000. Together with the good mechanical properties resulting from an ordered porous structure, reinforcement also came from particles stacking on top of each other, leading to a strong skeleton and excellent shape stability. The skeleton also formed a transfer network through the matrix that significantly increased the thermal conductivity reaching up to 1.34 W/m⋅K (538.1% higher than pure PEG 8000); this could also have increased the solar–thermal conversion efficiency to 95.2%. Another PVA-PEG-based AG-PCM composite was reported by Zhu et al. [51]. The four-step crosslinking, bidirectional freezing, freeze-drying, and vacuum impregnation fabrication process (Figure 8) led to obtaining bacterial cellulose/PVA/MXene AGs impregnated with PEG 20000. The material exhibited a photo-thermal conversion efficiency of 76.91% with 96.3% PCM loading (obtained because of the aligned pore structure of the AG) together with improved thermal conductivity of 0.768 W/mK (184% higher than PEG). The conversion effect may be attributed to the MXene, which induces coherent charge oscillations by resonant photons in the presence of incident photons at the same intrinsic frequency as the electrons, resulting in the generation of hot electrons. Then, the conjugated structures provide good transport channels for photoinduced carriers to accelerate electron migration through the fast conduction of electrons and holes. This leads electrons to an excited state, and then when they come back from it, they generate heat which is absorbed by the PCM undergoing a phase change. Weng et al. [52] used MXene as a light-to-heat conversion additive in cellulose AG-PEG 2000 composites prepared in situ by the freeze-casting method. As in previous works, MXene played a crucial role in improving thermal conductivity (0.4 W/m⋅K), which also facilitated the light-to-heat conversion efficiency reaching 91.6% and the latent heat reaching 183 J/g.

Liu et al. [53] modified a sargasso-delivered CNF-based AG with CNTs impregnated with PEG 4000. The CNF/CNT AGs were ideal substrate materials for PCMs due to their light absorption, electrical and thermal transfer network, and regular three-dimensional microporous structure leading to 85.6% photo-thermal energy storage efficiency and excellent cycling performance within 50 heating cycles, obtaining high latent heat (158.3 J/g) and low heat loss (0.75%). Another work focused on biomass-delivered xanthan gum polyimide (PI) TiO_2_ carbonized AGs by Sun et al. [54] impregnated with PEG 6000, which achieved a remarkable 94.23% light–thermal conversion and storage efficiency and improved thermal conductivity by 228% to 0.82 W/m⋅K (compared to PEG). The soft xanthan gum enables the stabilization of PI, which provides the rigid carbon framework skeleton and possesses high porosity and good thermal stability. XG helped to obtain a homogeneous dispersion of TiO_2_ in the network structure due to the super-stability and thickening effects.

Gui et al. [55] proposed carbonized syndiotactic polystyrene (sPS)/CNT/MXene hybrid AGs with an egg–box structure impregnated with PW. The CNT/MXene framework was initially prepared using freeze-casting. Subsequently, sPS was infused into the porous structure, followed by hyper-crosslinking and carbonization of sPS under an inert atmosphere. Due to the unique microstructure, the material was able to obtain a temperature of 123.7 °C in 120 s. After exposure to a solar simulator, the solid–liquid phase change of PW was indicated by a temperature plateau between 25 and 45 s of exposure. These types of composites are capable of synchronous EMI shielding and solar–thermal energy conversion due to conduction losses within the conductive network, polarization losses due to interfacial polarization, and dielectric losses within hierarchical pores.

Tang et al. [56] reported a high-energy-storage-density AG-PCM composite made from a phytic acid/melamine-modified Nb_2_CT_x_ MXene/delignified wood AG impregnated with n-docosane. The encapsulation ratio reached 91.6% and the latent heat stored was 219.5 J/g. The deposition of Nb_2_CT_x_ MXene enables the achievement of a solar-thermal conversion efficiency of 89.5%, reaching a temperature up to 103.2 °C in 300 s.

The examples provided possible ways to prepare AG-PCM combinations to obtain the most effective solar–thermal conversion performance, making the final material product functional and with synergistically improved properties. Those types of materials have a wide range of applications not only in the field of solar energy harvesting equipment but also in electronics, buildings, wearables, medical therapy, etc.

### 5.3. Solar–Thermal–Electric Conversion

The solar–thermal–electric conversion is mainly ascribed to two mechanisms: solar–thermal conversion (described in Section 5.2) and conversion of thermal energy into electrical energy ascribed to the Seebeck effect. Usually, solar thermoelectric generator systems (STEGs) are combined with materials with solar–thermal conversion ability to make the solar–thermal–electric conversion possible. The efficiency of the solar–thermal–electric conversion with STEGs ${(\eta }_{STEG})$ can be calculated using Formula (3) [58]:

(3) $$\eta_{STEG}=\frac{P_{0}}{P_{t}}\cdot 100\%$$

where $P_{0}$ and $P_{t}$ are total energy irradiated on the material and the output power by a STEG system, respectively.

**Figure 9:**
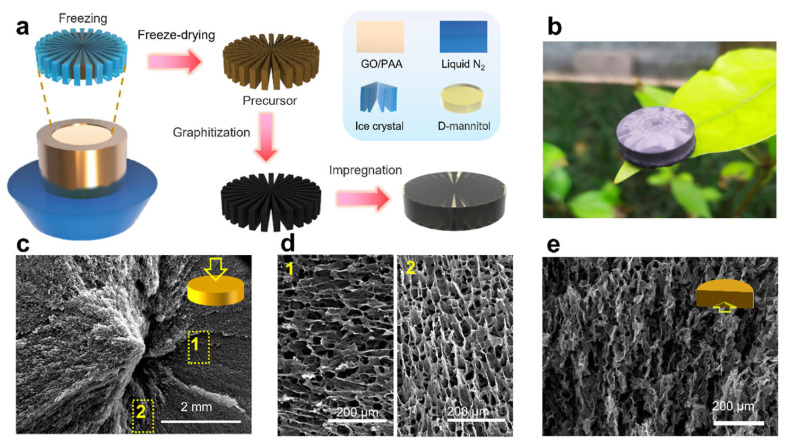
Structural design of the DM/graphene PCM. (**a**) Schematic preparation processes of the structured DM/graphene composites. (**b**) Digital photo of a graphitized GO/PI AG placed at the tip of a tree leaf. (**c**) SEM image exhibiting the top view of the graphitized GO/PI skeleton. (**d**) Enlarged details of the graphitized GO/PI skeleton in (**c**). (**e**) SEM image exhibiting the main view of the graphitized GO/PI skeleton. Reprinted with permission from the American Chemical Society [58].

Shu et al. [57] reported an AG-PCM composite of high-quality anisotropic graphene AG constructed to accommodate PW and impart improved thermal conduction, stable shape, and efficient solar–thermal conversion. The graphene AG was obtained by unidirectional freezing, freeze-drying, carbonization at 1000 °C, and graphitization at 2800 °C from pre-oxidized polyacrylonitrile and GO. The high-quality graphene network provides high thermal conductivity, shape stabilization, and sufficient enthalpy retention together with eminent solar light-absorbing capacity. The thermal conductivity reached 4.36 W/m⋅K. The output voltage under 5 kW/m^2^ irradiation reached 1181 mV and a current of 83.5 mA. Other work by Lin et al. [58] presents D-mannitol–graphene phase-change composites with structured conformation and thermal pathways (Figure 9). The composite implemented in the STEG was able to continuously collect clean solar energy and produce electricity based on the Seebeck effect, with an output power density of 784.92 W/m^2^, an ηSTEG up to 2.40%, and a voltage output of 3.21 V. Du et al. [60] proposed a dopamine-decorated Ti_3_C_2_T_x_ MXene/CNF AG impregnated with erythritol. The introduction of MXene sheets led to a solar–thermal conversion storage efficiency improvement of up to 88.9%, enabled the solar–thermal–electricity conversion, and reached a 0.63 V voltage output under 250 mW/cm^2^ irradiation intensity, which was also a result of the improved thermal conductivity (69% higher than that of pure PCM) with a value of 0.463 W/m⋅K.

Guo et al. [61] introduce a novel MoS_2_/montmorillonite hybrid AG impregnated with PEG 6000. The composite was prepared through freeze-drying and vacuum impregnation methods. The latent heat achieved was 169.16 J/g followed by a 96.47% efficiency of solar–thermal conversion heat storage because the addition of MoS_2_ significantly enhanced the light absorption performance. The output voltage was 458 mV under 2 kW/m^2^ light. Han et al. [59] obtained a functional AG-PCM composite from PPy hydrogel and a freeze-drying process followed by vacuum impregnation with PEG 6000. The material exhibited a high energy storage density of 142.4 J/g with an 86% efficiency of converting the solar light into heat. The composite was able to produce a 318 mV voltage output under a 250 mW/cm^2^ light radiation power density. The properties could be ascribed to the PPy due to its broad absorption spectrum range, high solar–thermal conversion efficiency, and high conductivity.

### 5.4. Magnetic–Thermal Conversion

Magnetic energy can be converted into heat if magnetic materials exhibit magnetic thermal coupling when subjected to the influence of an external alternating magnetic field, resulting in the production of magnetic–thermal effects. This relates to the reconfiguration of magnetic moments or changes in the microstructure of magnetic material that result in an increase in the material temperature.

The heat in magnetic–thermal conversion can be generated through Néel or Brownian relaxation in superparamagnetic materials when an alternating magnetic field is generated through the periodic electron motion in an alternating current [82]. Organic PCMs do not undergo this mechanism themselves, and they require some modifications with magnetic materials.

The magnetic–thermal conversion in PCM composites involves a few steps. First, an external magnetic field should be applied to the composite. The magnetic moments or domains in the magnetic material components of a composite reconfigure from one ordered magnetic state to another. In this step, the rotation of magnetic moments or movement of the magnetic domain consumes energy, which is why the material absorbs it, resulting in increased temperature, which continues until the equilibrium state is reached again. The PCM undergoes a phase change and can release heat during it. Magnetic–thermal conversion could be applied in thermomechanical systems, power generation, heating or cooling therapies, magnetic energy utilization, magnetocaloric diagnosis, thermal management/electromagnetic stealth of electronics, etc. [68].

The most common way to enhance the magnetic–thermal conversion abilities of AG-PCM composites is the addition of Fe_3_O_4_ particles. Song et al. [62] reported a composite of AG obtained from the in situ formation of Fe_3_O_4_ nanoparticles and the synchronous carbonization of kapok fibers impregnated with LA. The latent heat of the sample at ~93% PCM loading reached 161.7 J/g. The material was able to dissipate 98.2% of microwaves because of homogeneous anchored Fe_3_O_4_ nanoparticles, and the solar conversion efficiency reached 95% at 700 W/m^2^ and 73% at 1000 W/m^2^ for the AG samples prepared in 10 g/mL of Fe_3_O_4_ solution. In other work by Shen et al. [63], they prepared biomass-delivered AGs from a lignin GO suspension and thermal annealing at 900 °C. The AGs were impregnated via a vacuum impregnation process with Fe_3_O_4_ dispersed in PEG in different ratios. The AGs also exhibited high PCM encapsulation ability (load ratio 97.5%) with a melting enthalpy of 156.32 J/g and improved thermal conductivity of 281.1 S/m. The material also exhibited high light/electro-thermal conversion together with electromagnetic interference shielding at a magnetic field of 200 A/m of 53.83 dB at 7 wt% Fe_3_O_4_ in PEG samples. The magnetic-to-thermal conversion of composite samples in an alternating magnetic field was achieved by induced currents called eddy currents and hysteresis loss. The addition of Fe_3_O_4_ caused faster heating compared to samples with pure PEG because more heat is generated by hysteresis loss. The samples could reach up to 150 °C within 81 s for 7 wt% of Fe_3_O_4_ in PEG samples, which is a very fast result for a relatively low magnetic field. One way to calculate the conversion efficiency of the magnetic–thermal conversion (E) in materials with Fe_3_O_4_ is to use Equation (4) [83]:

(4) $$E=\frac{m_{s}}{m_{n}}\;C_{P}\frac{∆T}{∆t}$$

where $m_{s}$ and $m_{n}$ are the mass of the composites and NPs (Fe_3_O_4_), respectively. $C_{P}$ is associated with the specific heat capacity of the composite, and T and t are temperature and time.

Jin et al. [64] introduced a hybrid AG based on a Fe_3_O_4_-functionalized κ-carrageenan/melanin matrix impregnated with n-docosane. At 94.6% encapsulation of PCM, the composite reached 246.9 J/g. The samples exhibited low remanence magnetization and coercive force, which is typical for supermagnetic behavior. In an alternating magnetic field with a working frequency of 156 kHz, the sample with the highest Fe_3_O_4_-to-κ-carrageenan ratio (20:7) was able to reach 90 °C in 20 s compared to the lowest ratio (1:7) and only increased by 6 °C in 479 s.

Tao et al. [48] performed another study on carbonized hollow kapok fibers, functionalized with PPy and Fe_3_O_4_ via the coprecipitation method, and an AG impregnated with PW [65]. The material exhibited extremely low magnetic retentivity and coercivity, which confirmed the typical superparamagnetic behavior. The material was exposed to an alternating magnetic field (parameters: 2 A, 1.3 MHz, 6 kW, 1345 s). The material was able to reach 45 °C from room temperature in 100 s, then slowed down due to the phase transition within the PCM, and then accelerated and reached a stable temperature of 85 °C. In addition, the material possessed additional solar–thermal conversion ability with an efficiency of 90%.

**Figure 10:**
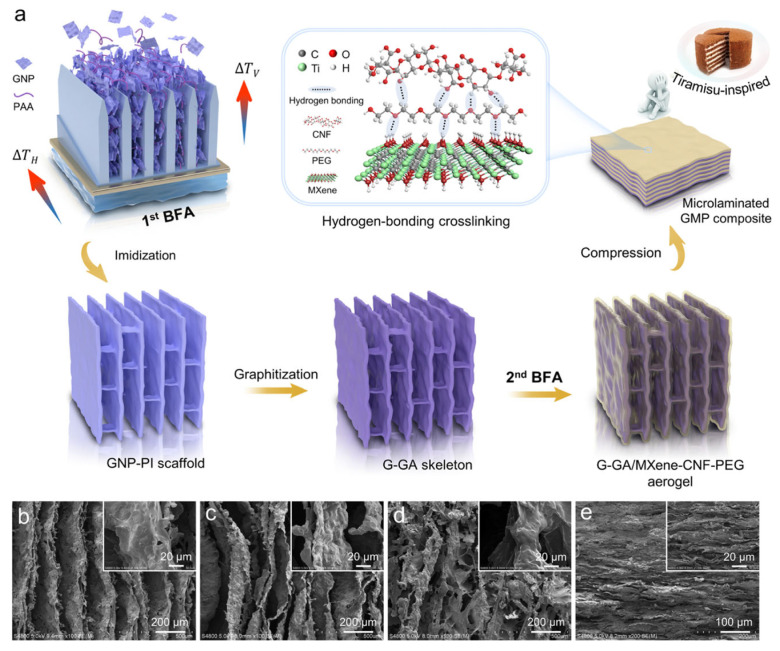
(**a**) Scheme illustrating the manufacturing route of tiramisu-like GMP composite. SEM images of (**b**) GNP-PI scaffold, (**c**) G-GA skeleton, (**d**) G-GA/MXene-CNF-PEG AG, and (**e**) micro-laminated GMP composite. Reprinted with permission from Elsevier [66].

Electromagnetic interference (EMI) shielding materials are employed to abate electromagnetic radiation by absorbing electromagnetic waves and further converting them into considerable heat, which may be beneficial for microelectronics. Hu et al. [66] prepared a tiramisu-like phase-change nanocomposite (Figure 10) from a graphitized graphene array/MXene/CNF-PEG AG obtained via bidirectional freezing assembly. The PEG was encapsulated onto the microlayer surfaces of the graphitized graphene array by a crosslinking network created by the hydrogen bond actions of MXene-CNF. The thermal conductivity of the samples reached 34.05 W m^−1^ K^−1^, a shielding effectiveness of 87.4 dB, and a solar–thermal conversion efficiency of 90.1%.

### 5.5. Acoustic–Thermal Conversion

Acoustic–thermal conversion can occur through the ultrasound thermal effect when a mechanical wave that possesses high energy and high penetration force is transformed into thermal energy. When the sound wave propagates through a solid medium, it can cause internal friction, which is a result of the relative motion between medium molecules. The wave energy that causes the internal friction is converted into heat. There is also a heat exchange between the dense and sparse parts of the medium, as well as heat conduction, which leads to acoustic attenuation and heat generation [11].

The main mechanisms for heat generation and melting enhancement are related to ultrasonic vibrations that occur together with agitation, acoustic streaming, cavitation, and oscillating fluid motion. Additionally, heat transfer can be enhanced by the acoustic streaming effect of the ultrasonic field, which creates vortical flows in the heat transfer medium [84]. On the other hand, the cavitation effect creates bubbles that can be transferred through the liquid and enhance the heat transfer [85].

Following this mechanism, Liu et al. [67] developed an AG-PCM composite to convert sonic energy into thermal energy. The material, based on PEG 6000 as a PCM and a GO framework functionalized by Fe_3_O_4_ nanoparticles, was prepared by in situ forming a crosslinked hydrogel in the first step and freeze-drying it to obtain the final composite. The unique structure of the composite, especially the GO-enriched material, not only provides enhanced thermal conductivity but also provides the ability to absorb sonic waves. The PEG/Fe_3_O_4_-GO composite showed a high thermal energy storage density (173.7 J/g). The acoustic–thermal conversion test showed that with increasing sonication time, the temperature of the material increased. The temperature of PEG/Fe_3_O_4_-GO increased rapidly after exposing the material to acoustic waves at 1200 Hz, reaching 74 °C, which was ascribed to the scattering, reflection, and micro-vibrations that occurred within the microstructure of the Fe_3_O_4_-GO network.

Functional AG-PCM composites with the ability to convert mechanical–acoustic waves into heat may be used in the medical field of ultrasonic therapy, a building sector for sound isolation, or as a material for noise mitigation or super-wave hyperthermia fields. Further research may be needed to evaluate other types of AG modification for this type of conversion, which is less studied in comparison to the other ones.

## 6. AG-PCM Additional Properties for Functional Composites

### 6.1. Thermal Conductivity Enhancement

One of the main disadvantages of many solid–liquid PCMs is their low thermal conductivity. Various types of AGs, including carbon, metallic, or boron nitride, can be used to improve this parameter, which is important from the point of view of many PCM applications [86].

Wan and co-workers [87] prepared a BNNSs-g/CNF/PEG multifunctional composite. The functionalized boron nitride nanosheets were further crosslinked with hydroxyl-rich cellulose nanofibers to obtain porous AG. Its role was not only to stabilize the shape and prevent leakage but also to improve the thermal conductivity of the PCM. The PEG/BNNSs-g/CNF composite exhibited a high phase-change enthalpy of 150.1 J/g, which is approximately 94% of that of pure PEG, as well as high thermal diffusivity, about 4.5 times higher than that of pure PEG. In the end, when the obtained composite was compressed beyond the melting point of PEG, it remained stable without any leakage.

**Figure 11:**
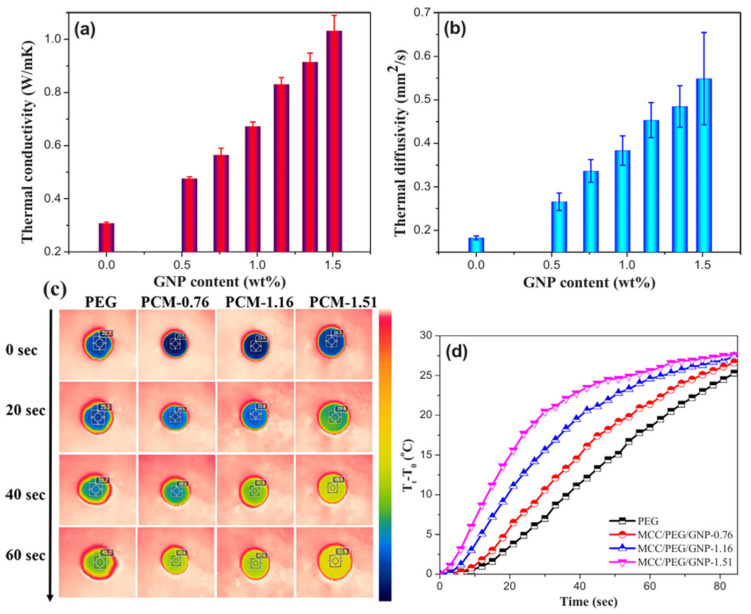
Effects of GNP content on the thermal conductivity (**a**) and thermal diffusion coefficient (**b**) of the different samples, (**c**,**d**) showing the temperature changes of the sample surface with increasing time when it was placed on a hot stage set at 80 °C. Reprinted with permission from Elsevier [88].

Wei et al. [88] proposed a PEG composite with microcrystalline cellulose (MCC)/graphene nanoplatelets (GNPs) in an AG. The highly anisotropic MCC/GNP AGs were obtained by solution compounding, gelling, solvent exchanging, and freeze-drying in the end. PEG impregnation took place in a vacuum oven. The tested composite with 1.51 wt% GNP content was characterized by high thermal conductivity (1.03 W/m⋅K, over 3 times higher than the MCC/PEG composite). Importantly, the value of the phase transformation enthalpy did not decrease significantly, as it constituted up to 99.84% of the value for pure PEG. These composite PCMs demonstrate outstanding encapsulation ability and mechanical stability at temperatures above the melting point of PEG, resulting from the formation of an orientated structure and a 3D segregated structure. Figure 11 presents the effects of GNP content on different composite properties.

Graphene AG (GAG) was used to shape the support and enhance the thermal conductivity of octadecanoid acid (OA) by Zhong et al. [89]. GAG was formed from GO sheets through a hydrothermal reaction. Then, stearic acid was infiltrated into an AG via a vacuum oven to obtain a GAG/OA composite. The obtained composite was characterized by a high heat storage capacity of 181.8 J/g, which was nearly 98% of the value for pure octadecanoid acid. The thermal conductivity of the GAG/OA composite increased around 14 times in comparison with OA (from 0.184 W/m⋅K to 2.635 W/m⋅K).

Cheng et al. [90] used a one-step in situ synthesis method to prepare SSPCM, formed by incorporating PEG into a chemically crosslinked cellulose nanocrystal (CNC) AG. The obtained composite exhibited a high phase-change enthalpy of 145.8 J/g (80% of the value for pure PEG) and remarkable cyclic reversibility after 100 thermal cycles. The shape stabilization was also very satisfactory as the SSPCM maintained its original shape without any leakage even when compressed at the melting point of PEG. The thermal conductivity of the composite increased by almost 25% (from 0.34 W/m⋅K to 0.42 W/m⋅K) compared to pure PEG due to the high CNC value.

Tian et al. [91] prepared composites based on CNT–graphene AGs (CGA) with infiltrated PW. Carbon AGs were produced directly by the modified hydrothermal method, cryodesiccation, and then subjected to the calcination process. The phase-change parameters of the obtained composite did not change significantly compared to those of the PW. The phase transition was at 48.08 °C and the latent heat value was around 222 J/g. The leakage test confirmed the shape stability of the composite up to the melting point of pure PW; above 50 °C, the material began to leak (12% leakage rate at 60 °C). The tested composite with 2 wt% of the carbon AG was characterized by high thermal conductivity (2.182 W/m⋅K, which is over 10 times higher than for pure PW).

Copper nanowire AGs (CuNW-AGs) infiltrated with PW composites were tested by Zhang [92]. CuNW-AGs were prepared based on the “bubble-controlled assembly” mechanism, and 2 wt% of CuNW-AGs was tested in a phase-change material composite. An over 30% increase from 0.21 W/m⋅K to 0.28 W/m⋅K of thermal conductivity characterized the obtained composite compared to pure PW. Additionally, the electrical conductivity rose from 0 to 3.03 S/m for the CuNW-AG-PW composite. The phase-transition enthalpy of the obtained composite decreased by only 1% (to 173.2 J/g) compared to pure PW, which is crucial for thermal energy storage materials. A shape stabilization test was also carried out at 70 °C, during which there was only a slight PW leak, confirming the AG’s role as supporting material.

Overall, the enhancement of thermal conductivity is one of the main goals in the case of organic PCMs. Boosting the thermal conductivity of AG-PCM composites improves the response time; consequently, those composites may be used, e.g., in thermal management and energy conversion. For this purpose, most often, carbon-based AGs are used. However, in some cases, the thermal conductivity improvement may be limited due to the significant interfacial thermal resistance between PCMs and conductive nanofillers [93]. Table 2 lists the properties of various AG-PCM composites with improved thermal conductivity.

### 6.2. Mechanical Flexibility

Apart from shape stabilization and leakage prevention, AGs can also significantly improve the mechanical properties of PCMs. We can distinguish graphene AGs, cellulose AGs, and polymer AGs among the flexible AGs. Flexible PCMs can endure some deformation and ensure better contact with intricate structural surfaces, thereby enhancing the thermal management of complex device surfaces [95,96].

Cai et al. [97] proposed an ultra-light and flexible graphene AG-based form-stable phase-change material for energy storage and conversion. Light graphene AG (LG-AG) was prepared using sodium dodecyl sulfate as a microbubble template, natural latex, and graphene. The prepared LG-AG was then used as a support and functional material for PW encapsulation during the formation of the PCM composite. Additionally, styrene-b-(ethylene-co-butylene)-b-styrene was incorporated into the composite to prevent the leakage of PW. The obtained PCM composite was characterized by an encapsulation efficiency of 90 wt%, a high phase-change enthalpy of 212.4 J/g, and great thermal reliability after 200 thermal cycles. Additionally, the PCM composite presented good electro/photo-thermal conversion due to the excellent photo/electric effect of graphene AG.

Lyu and co-workers [98] reported form-stable PW/PTFE/SiO_2_ phase-change AG films. PW (62.8 wt%) was infiltrated into the PTFE/SiO_2_ AG film. Because of their extremely low density, capillary forces, and porous architecture, AG films can keep a large amount of PW, thereby effectively preventing any leakage. The phase-change AG composite film was characterized by a large phase-transition enthalpy of 128 J/g and extraordinary cyclic reversibility. The obtained PTFE/SiO_2_ AG in the form of a thin film (185 μm) gave the composite additional properties such as favorable flexibility and low surface density. Additionally, the production of films from phase-change composites provided various responses to thermal stimuli, such as changes in wettability, mechanical properties, and transmittance.

**Figure 12:**
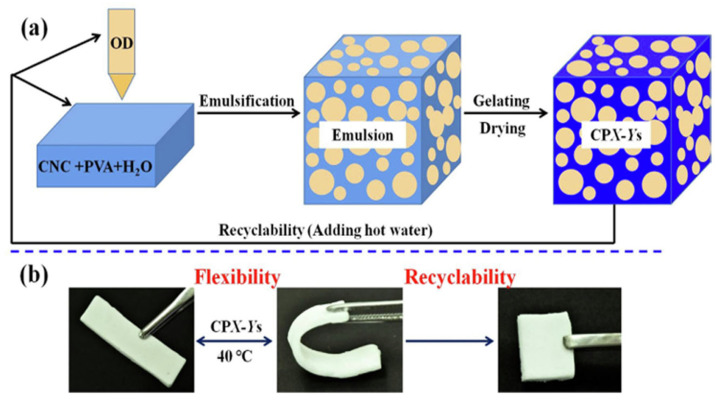
Scheme illustrating (**a**) the fabrication of emulsion-based, OD-encapsulated AG composites and (**b**) their flexibility and recyclability. Reprinted with permission from Elsevier [96].

Zhao et al. [96] presented AG composites from cellulose nanocrystal-stabilized, octadecane-encapsulated Pickering emulsions solidified using physical gelation. Additionally, the introduction of PVA significantly limited the leakage of the encapsulated octadecane. The resulting composites exhibited similar heat capacity (up to 253 J/g) to bulk octadecane, and demonstrated high reusability, showing no significant deterioration in heat capacity even after 100 thermal cycles. The AG composites showed controlled external shapes and exhibited flexibility at temperatures above the melting point of the encapsulated octadecane, which was 30 °C. They also showed robust compressive strength, enduring up to 70% compressive strain without fracturing. Furthermore, the composites demonstrated recyclability when dissolved in hot water to create emulsions, which could then be freeze-dried to re-form the AG composites. Figure 12 presents the fabrication and properties of AG composites.

To conclude, flexible AGs are intriguing candidates for PCM shape stabilization. Cellulose and polymer AG-PCM composites, known for their outstanding mechanical properties and flexibility, also demonstrate shape memory functionality. Flexible PCMs can endure some degree of deformation and ensure closer contact with complex structural surfaces, thereby enhancing the thermal management efficiency of complex device surfaces. New flexible AG-PCM composites present significant application potential in the thermal management of wearable devices, shape-memory materials, and thermoregulating textiles.

### 6.3. Thermal Protection/Insulation Properties

Although the first thing that comes to our mind is the improvement of thermal conductivity, in the case of phase-change materials, reducing this parameter is often equally important. During thermal protection, reducing the thermal conductivity of the material is crucial. One of the basic methods of reducing thermal conductivity is the application of porous substrates with low thermal conductivity, including popular silica AGs [17,99].

Zhang and co-workers [100] tested a novel PCM composite with good insulation properties. The n-octadecanol was infiltrated into a GO/silica hybrid AG with a porous structure that was synthesized using the sol–gel method. AG pore structure analysis indicated that the addition of GO to the silica sol can prevent the pores from collapsing during drying to a certain extent. Therefore, the volume shrinkage can be reduced. The DSC results showed that a composite with 0.5 wt% GO had the best thermal properties (145.6 J/g heat storage capacity). The thermal conductivity of the composite (0.0808 W/m⋅K) decreased by 73% compared to pure octadecanol. Furthermore, the temperature of the described composite can reach its melting point when exposed to irradiation, demonstrating its effective light-to-thermal conversion capability.

**Figure 13:**
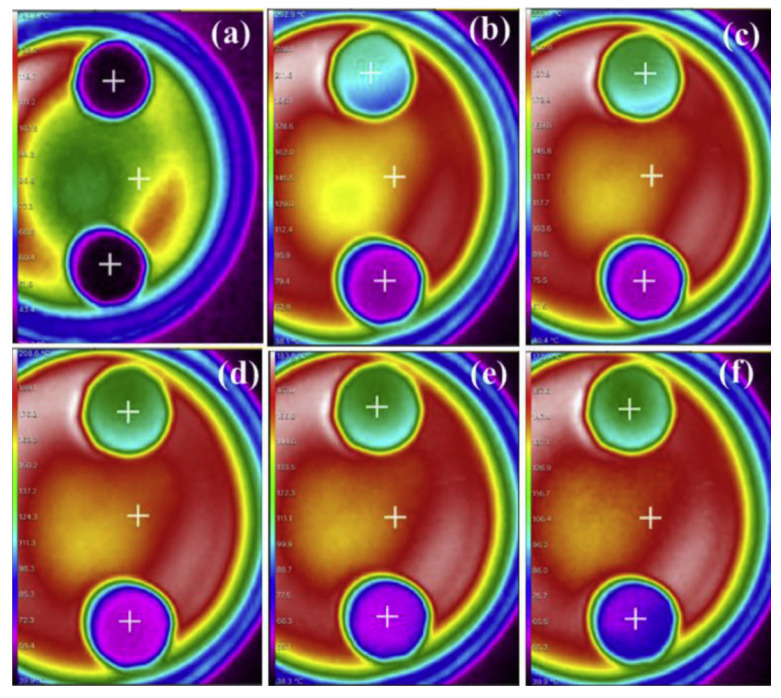
Infrared thermal images of the (upper) CSA and (lower) CSA/PA samples with varying times: (**a**) 0 min, (**b**) 2 min, (**c**) 5 min, (**d**) 7 min, (**e**) 10 min, and (**f**) 13 min. Reprinted with permission from Elsevier [101].

Ding et al. [101] presented PCM composites using palmitic acid (PA) as the PCM and a 3D porous carbon/silica composite AG (CS-AG) as the porous supporting material. PA molecules were effectively combined with CS-AG through capillary forces and surface tension. The leakage test indicated that the CS-AG/PA composite exhibits great shape stabilization properties. The melting latent heat of the composite was 187.7 J/g, which is 88% of the value for pure PA. Additionally, after 50 thermal cycling tests, the latent heat of the CS-AG/PA composite remained almost unchanged, indicating its excellent thermal stability. The composite was also characterized by relatively low thermal conductivity (0.179 W/m⋅K), which is beneficial for good thermal insulation properties. Further thermal imaging camera tests were also performed to evaluate short-term thermal insulation (Figure 13). The initial surface temperatures of the two samples were quite similar (Figure 13a). Over time, the surface temperatures of both samples rose; however, the temperature increase in the CS-AG/PA sample was significantly slower than that in the CS-AG sample, which can be attributed to the phase-change process occurring at higher temperatures.

**Figure 14:**
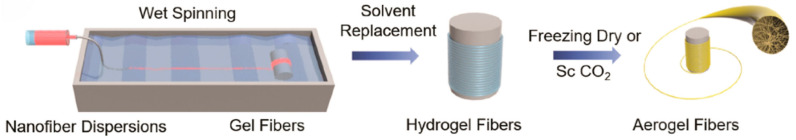
Schematic description of the fabrication of KNF AG fibers. Reprinted with permission from American Chemical Society [102].

Liu et al. [102] prepared phase-change AG fibers by incorporating PEG into Kevlar (KNF) AG fibers. The AG fibers exhibited great potential for heat insulation, with the thermal conductivity of the fabricated AG fiber mats measured at only 0.04 W/m⋅K. The phase-change fibers demonstrated a significant energy storage capacity, as their phase-change enthalpy was 162 J/g. Figure 14 presents the KNF AG fibers’ fabrication process.

As stated above, in some cases, low thermal conductivity may be desirable. AGs with low thermal conductivity limit heat flow in the PCM; thus, AG-PCM composites with low thermal conductivity can be used in heat insulation and preservation. Silica AG is most commonly used for this purpose, as it is characterized by extremely low thermal conductivity. The properties of different AG-PCM composites with thermal insulation are presented in Table 3.

### 6.4. Flame Retardancy

Poor flame retardancy is a huge drawback of organic PCMs, which restricts their widespread use in various expanding industries, including energy-efficient buildings and electronic vehicles. To reduce flammability and improve fire safety, various approaches have been developed. The most frequently used is the incorporation of flame retardants including intumescent FR (melamine, APP, PER), halogenated alkanes, heat absorbers, and synergists (nano clays, metallic nanoparticles) [105].

**Figure 15:**
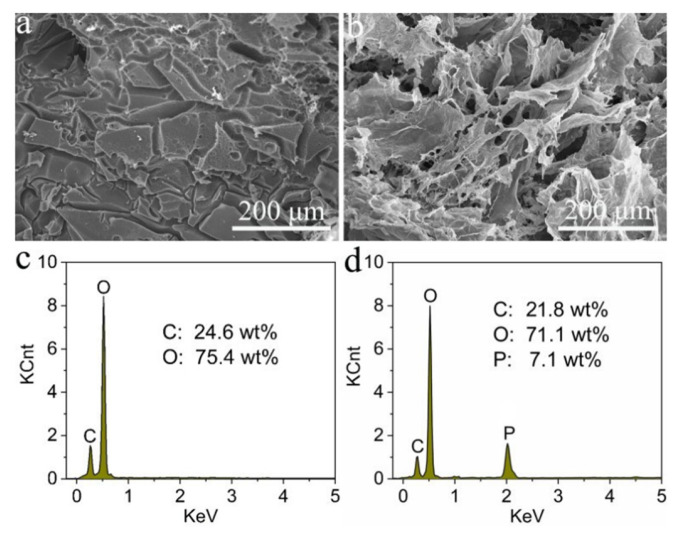
SEM images and EDS spectra of residual chars for CBPCM with no BP nanosheets (**a**,**c**) and CBPCM with 24 wt% of BP nanosheets (**b**,**d**). Reprinted with permission from Royal Society of Chemistry [106].

Du et al. [106] proposed novel form-stable PCM composites based on cellulose nanofiber (CNF), 2D-layered black phosphorus (BP) nanosheets, and n-octacosane by impregnating alkane/PW into the CNF/BP AGs (CBPCMs). The obtained composites were characterized by high phase-change enthalpies ranging from 247.0 to 251.6 J/g. The tests also indicated a high loading rate of n-octacosane of nearly 3000%, resulting from the BP nanosheets effectively improving the hydrophobicity of the hybrid AG. The decrease in the rate of heat release and total heat release and the increase in the char yield and LOI value confirm the enhancement of the flame retardancy of the CBPCM composites. Moreover, the addition of BP nanosheets to the AG significantly increased thermal conductivity by 89% and solar–thermal conversion and storage efficiency by up to 87.6%. Figure 15 shows SEM and EDS results of residual chars for CBPCM.

Luo et al. [107] tested multifunctional composites based on MXene AG infiltrated with stearyl alcohol modified by phosphorus-containing molecules (PSMs). Based on research results, MXene AGs are a great support material for PCMs’ shape stabilization and leakage prevention. Moreover, the combination of MXenes and phosphorus improves the flame retardancy of the obtained composites, which is due to the decrease in the peak heat release rate by 42.8% and total heat release by 32.1%, as well as an increase in char residue at 800 °C. The presence of MXene in composites results in a significant thermal conductivity increase of up to 0.486 W/m⋅K. The values of relative enthalpy efficiency are greater than 95%, which means that the thermal properties of the composites are very satisfactory.

Li and co-workers [108] prepared composites consisting of stearyl alcohol (SAL) chemically bonded with phosphorus oxychloride infiltrated into the SWCNT AG (PCMF). The normalized enthalpy value of PCMFs was close to the value of the modified alcohol, which was about 84% of the pure SAL value. Microscale combustion calorimetry tests showed that the peak of the heat release rate and total heat release of the modified SAL were reduced by over 20% compared to pure alcohol. After encapsulating the modified SAL into the SWCNT AG, the mentioned parameters of the prepared composites can be further reduced due to the synergistic effect between the phosphorus group and the SWCNTs. Additionally, the SWCNT AG significantly enhanced the electrical and thermal conductivity of the PCMF.

Novel multifunctional PEG-based composite phase-change materials were obtained by Zhou et al. [109]. PVA AG was used for shape stabilization, while APP (ammonium polyphosphate), boron nitride, and long-afterglow luminescent particles were used for further modifications. The composite phase-change material exhibited an excellent phase-change enthalpy of 163.9 J/g, which was 91.1% of the value for pure PEG. The leakage test confirmed its shape stability as the composite was heated at 80 °C for 1 h with no leakage. Additionally, the thermal conductivity increased significantly to 0.32 W/m⋅K. The PCM composite exhibited outstanding thermal stability and flame retardancy, with the maximum decomposition rate and the maximum heat release rate reduced by 47% and 34.1%, respectively, compared to pure PCM. Additionally, the composite PCMs achieved light energy storage alongside thermal energy management and utilization. They could absorb and store ultraviolet and visible light during the day, and then gradually release fluorescence over an extended period in the dark.

Cao et al. [110] proposed silica-based AGs encapsulating organic composite phase-change materials for building thermal management. The aim was, among other things, to improve the flammability of the organic PCM. The mixture of methyl palmitate and an inorganic eutectic hydrate salt modified with sodium CMC was used as a PCM, and then it was vacuum-impregnated into silica AG. The obtained composite was characterized by a high latent heat value of 174.1 J/g at 22.9 C. Even after 100 cold/thermal cycles, its thermal properties remained largely unchanged, demonstrating its strong cycle stability. The silica AG resulted in good shape stability and significant flame retardancy improvement. The composite could not be ignited even when the oxygen concentration was raised to 100%, demonstrating its exceptional flame-retardant performance.

To summarize, AG-PCM composite’s flame retardancy improvement may be connected to the introduction of flame-retardant fillers into the PCM or AG matrix. Even the AG (MXene) can act as a flame retardant. Flame-retardant AG-PCM composites find their applications in buildings, batteries, or medical devices. To describe materials’ flammability, total heat release (total HR), peak heat release rate (peak HRR), limiting oxygen index (LOI), and char residue are usually used. Table 4 presents the properties of AG-PCM composites with lower flammability.

## 7. Conclusions and Future Directions

AG-PCM composites are a large emerging group of materials that can be used for multiple applications, starting from energy, through medical, space, buildings, army systems, etc. The abundant applications were not only from the properties related to latent heat storage but also an outcome of properly designed and functionalized AGs which together could form multifunctional materials. Especially today, environmental concerns are pushing research into highly sustainable and multifunctional materials that could save, store, or harvest energy together with additional superfunctionality, making them even more efficient or sustainable. Thus, AG-PCM composites are the perfect solution to fulfill those requirements.

Materials with conversion-related functionalities have emerged strongly in recent years. The main focus is solar–thermal conversion performance, which is usually attributed, as has been presented in this article, to carbon additives which are one of the best conversion enhancers among all materials, like CNTs, GO, graphene, etc., which are deposited or directly form the core of AGs for double enhancement of PCM, shape stability, and conversion. However, more research is still needed to evaluate which type of addition (directly deposited on PCM or deposited on the AG) would be a better option for tested performance and efficiency. Also, in terms of solar–thermal–electric conversion materials, there is a big knowledge gap on how to provide an “all-in-one” material that could work without external converters. However, the magnetic and electromagnetic conversion field of AG-PCM AGs is evolving and proposing very promising solutions based on magnetite and the newly emerging MXene material, which still need to be further studied in terms of larger-scale production methods. However, those additives are capable not only of providing the desired conversion but also of significantly enhancing the thermal conductivity of composites or providing them with additional conversion ability, making them multifunctional.

Other multifunctional AG-PCM composites focus not only on thermal-based properties but also on some other characteristics that may expand the area of their application. Much research pays attention to the thermal conductivity of the composite, which, depending on the application, may be as high or as low as possible. MXene AGs, graphene AGs, metal AGs, and BN AGs are most commonly used for AG-PCM composite thermal conductivity improvement. On the other hand, silica AGs and polymer AGs are utilized for thermal insulation. Polymer AGs, BN AGs, and carbon AGs ensure the high flexibility of the obtained composites. Flame-retardant AG-PCM composites can be obtained by using MXene AGs and flame-retardant additives such as phosphorus-containing molecules. Therefore, the selection of appropriate components in the PCM-AG–additive system may allow for obtaining innovative, multifunctional composites for unusual applications.

The main trends observed by authors that may develop in the future are the following:

- The usage of biobased AGs or biomass-based AGs that may be more sustainable compared to the conventional ones.
- The combination of different nanoparticles and nanomaterials for effective enhancement of different types of properties for multifunctional usage.
- The development of MXene-based AGs not only based on carbides but also nitrides.
- The development of flexible AGs with increased flexibility.
- Further improvement of AG-PCM in electromagnetic shielding with multifunctional capabilities.
- Additional techniques are required to form phase-change AGs into 1D fibers or 2D films, resulting in enhanced properties for thermal management systems.
- To expand the area of application, it would be worth expanding research on the mechanical properties of various support structures.
- Inorganic PCMs characterized by high latent heat should also be tested, as they may prove to be an interesting direction of research.

## Figures and Tables

**Table 1:** Properties of AG-PCM composites with different conversion abilities.

Conversion Type	Aerogel	PCM	PCM Loading [%]	Tm [°C]	Latent Heat [J/g]	Conversion Efficiency [%]	Ref.
Electro-thermal	MOF-C/GO	LA	−	55	140	90	[40]
Electro/solar-thermal	rGO/cellulose sodium	LA/MA SEBS	99.7	−	124.6	82.3/96.5	[41]
	MF/GNT/CNT	n-octadecane	85.8%	38	239.12	−/95	[42]
	CNF/CNT/MXene	PW	−	50.5	133	−	[43]
	CMC-CNTs/PPy	PW	80.9	−	147.9	91.5	[44]
	GO/AgNPs	PW	98.34	53.72	133.86	87.12/92.62	[45]
	Carbonized plant straw	PEG 4000	97	75.2	189.4	−/92.3	[46]
	HNTs-graphene	PU	98.83	57.4	103.3	66.3/78.4	[47]
Solar-thermal	PVA/CNT	PEG 6000	−	59.2	140.4	89.6	[48]
	MNs/PVA/rGO	LA	98.5	42.3	191.2	91.85	[49]
	PVA/BNNs/PDA@TZnO	PEG 8000	91.1	63.23	139.0	95.2	[50]
	PVA/BC/MXene	PEG 20000	96.3	59.80	157.7	76.91	[51]
	Cellulose/MXene	PEG 2000	90	59.1	183	91.6	[52]
	CNF/CNT	PEG 4000	90	63.55	158.3	85.6	[53]
	Xanthan gum/PI/TiO_2_	PEG 6000	92	61.24	160.38	94.23	[54]
	PS/CNT/MXene	PW	79.1	60	158.1	−	[55]
	Nb_2_CT_x_ MXene/Delignified Wood	n-docosane	81.2	47.2	194.6	89.5	[56]
Solar-thermal-electric	OPAN/GO	PW	−	58.2	187.6	−	[57]
	Graphene	d-mannitol	−	168	199	2.4	[58]
	PPy	PEG 6000	−	63	142.4	−	[59]
	CNF/MXene	erythritol	−	125.3	330.6	−	[60]
	MoS_2_/montmorillonite	PEG 6000	−	59.55	168.98	−	[61]
Magnetic/solar-thermal	Carbonized kapok fiber/Fe_3_O_4_	LA	92.2	44.5	161.7	98.2/73	[62]
	Carbonized lignin/GO/Fe_3_O_4_	PEG 4000	91.3	57.2	149.19	−	[63]
	κ-carrageenan/melanin/Fe_3_O_4_	n-docosane	94.6	45.9	246.9	−/93.5	[64]
	Carbonized kapok fiber/PPy/Fe_3_O_4_	PW	88	51.3	161.4	−	[65]
	Graphitized graphene array/MXene/CNF	PEG 8000	−	60.24	179.4	−	[66]
Acoustic-thermal	GO/Fe_3_O_4_	PEG 6000	99.5	59.95	173.7	−	[67]

**Table 2:** Properties of AG-PCM composites with thermal conductivity enhancement.

PCM	Aerogel	Tm [°C]	Latent Heat [J/g]	PCM Thermal Conductivity [W/m⋅K]	Composite Thermal Conductivity [W/m⋅K]	Ref.
PEG	BNNSs-g/CNF	45.2	150.1	0.033 ^a^	0.148 ^a^	[87]
PEG	MCC/GNP	~70	182.6	0.31	1.03	[88]
octadecanoid acid	graphene	~56	181.8	0.184	2.635	[89]
PEG	cellulose nanocrystal	57.3	145.8	0.34	0.42	[90]
PW	CNT–graphene	48.08	222	0.208	2.182	[91]
PW	copper nanowire	~55	173.2	0.21	0.28	[92]
PW	boron nitride	46.9	183	0.2	0.29	[94]

^a^ thermal diffusivity [mm^2^/s].

**Table 3:** Properties of AG-PCM composites with thermal insulation.

PCM	Aerogel	Tm [°C]	Latent Heat [J/g]	PCM Thermal Conductivity [W/m⋅K]	Composite Thermal Conductivity [W/m⋅K]	Ref.
octadecanol	GO/silica	~60	145.6	0.3015	0.0808	[100]
palmitic acid	porous carbon/silica	55.71	187.7	0.170	0.179	[101]
PEG	Kevlar fibers	~60	162	0.04 ^b^	−	[102]
octadecanol	silica	60.13	127.73	0.25	0.12	[103]
PEG	silica	57.40	90.63	0.29	0.08	[103]
erythritol	silica	123.8	289.9	0.7–0.8 ^c^	0.31	[104]

^b^ Kevlar AG fiber value. ^c^ literature value.

**Table 4:** Properties of various AG-PCM composites with flame retardancy.

PCM	Aerogel	Additives	Tm [°C]	Latent Heat [J/g]	TotalHR [kJ/g]	PeakHRR [W/g]	Char Residue % (Temp.)	Ref.
n-octacosane	CNF	BP	65.7	248.8	42.88	728.1	3.51 (700)	[106]
stearyl alcohol	MXene	phosphorus oxychloride	79.2	120.1	61.4 MJ/m^2^	440.2 kW/m^2^	27.8 (800)	[107]
stearyl alcohol	SWCNT	phosphorus oxychloride	68.8	101.4	12.3	772.6	43.39 (800)	[108]
PEG	PVA	APP, BN	61.7	163.9	21.6	459.5	7.4 (600)	[109]
PEG	MXene/PI	-	62	167.9	21.4	529.3	3.3 (800)	[111]

## Data Availability

Not applicable.

## Abbreviations

AG	aerogel	MF	melamine foam
APP	ammonium polyphosphate	MNs	montmorillonite nanosheets
BBNs	boron nitride nanosheets	MOF-C	metal–organic frame—carbon
BN	boron nitride	NPs	nanoparticles
BP	black phosphorus	OA	octadecanoid acid
CMC	carboxymethylcellulose	PA	palmitic acid
CNC	cellulose nanocrystal	PCM	phase-change material
CNF	cellulose nanofibers	PDA	polydopamine
CNTs	carbon nanotubes	PEG	poly(ethylene glycol)
CS	carbon/silica	PPy	polypyrrole
CuNW	copper nanowire	PU	polyurethane
EDS	Energy-Dispersive Spectroscopy	PVA	poly(vinyl alcohol)
EMI	electromagnetic interference	PW	paraffin wax
GNPs	graphene nanoplatelets	rGO	reduced graphene oxide
GNTs	graphene nanoplatelets	SAL	stearyl alcohol
GO	graphene oxide	SEM	scanning electron microscope
HNTs	halloysite nanotubes	sPS	syndiotactic polystyrene
KNF	Kevlar	SSPCM	shape stabilization of phase-change materials
LA	lauric acid	STEG	solar thermoelectric generator systems
MA	myristic acid	TES	thermal energy storage
MCC	microcrystalline cellulose	T-ZnO	tetrapod zinc oxide

## References

[1] IEA (2022). World Energy Outlook 2022—Analysis. https://www.iea.org/reports/world-energy-outlook-2022.

[2] Li C., Yu H., Song Y., Liang H., Yan X. (2019). Preparation and characterization of PMMA/TiO_2_ hybrid shell microencapsulated PCMs for thermal energy storage. Energy.

[3] Lv P., Ding M., Liu C., Rao Z. (2019). Experimental investigation on thermal properties and thermal performance enhancement of octadecanol/expanded perlite form stable phase change materials for efficient thermal energy storage. Renew. Energy.

[4] Li M., Guo Q., Su Y. (2022). The thermal conductivity improvements of phase change materials using modified carbon nanotubes. Diam. Relat. Mater..

[5] Yu X.K., Tao Y.B., He Y., Lv Z.C. (2022). Temperature control performance of high thermal conductivity metal foam/paraffin composite phase change material: An experimental study. J. Energy Storage.

[6] Thapliyal P.C., Singh K. (2014). Aerogels as Promising Thermal Insulating Materials: An Overview. J. Mater..

[7] Bheekhun N., Abu Talib A.R., Hassan M.R. (2013). Aerogels in Aerospace: An Overview. Adv. Mater. Sci. Eng..

[8] Mao J., Iocozzia J., Huang J., Meng K., Lai Y., Lin Z. (2018). Graphene aerogels for efficient energy storage and conversion. Energy Environ. Sci..

[9] Mahmoudpour M., Dolatabadi J.E.-N., Hasanzadeh M., Soleymani J. (2021). Carbon-based aerogels for biomedical sensing: Advances toward designing the ideal sensor. Adv. Colloid Interface Sci..

[10] Liu P., Chen X., Li Y., Cheng P., Tang Z., Lv J., Aftab W., Wang G. (2022). Aerogels Meet Phase Change Materials: Fundamentals, Advances, and Beyond. ACS Nano.

[11] Kong X., Nie R., Yuan J. (2023). A review of shape stabilized aerogel-based phase change materials for preparation, classification and applications. Energy Built Environ..

[12] Abdul Khalil H.P.S., Bashir Yahya E., Jummaat F., Adnan A.S., Olaiya N.G., Rizal S., Abdullah C.K., Pasquini D., Thomas S. (2023). Biopolymers based aerogels: A review on revolutionary solutions for smart therapeutics delivery. Prog. Mater. Sci..

[13] Lee J.-H., Park S.-J. (2020). Recent advances in preparations and applications of carbon aerogels: A review. Carbon.

[14] Ganesamoorthy R., Vadivel V.K., Kumar R., Kushwaha O.S., Mamane H. (2021). Aerogels for water treatment: A review. J. Clean. Prod..

[15] Maleki H. (2016). Recent advances in aerogels for environmental remediation applications: A review. Chem. Eng. J..

[16] Singh P., Sharma R.K., Ansu A.K., Goyal R., Sarı A., Tyagi V.V. (2021). A comprehensive review on development of eutectic organic phase change materials and their composites for low and medium range thermal energy storage applications. Sol. Energy Mater. Sol. Cells.

[17] Yuan K., Shi J., Aftab W., Qin M., Usman A., Zhou F., Lv Y., Gao S., Zou R. (2020). Engineering the Thermal Conductivity of Functional Phase-Change Materials for Heat Energy Conversion, Storage, and Utilization. Adv. Funct. Mater..

[18] Wang X., Li W., Luo Z., Wang K., Shah S.P. (2022). A critical review on phase change materials (PCM) for sustainable and energy efficient building: Design, characteristic, performance and application. Energy Build..

[19] Aftab W., Usman A., Shi J., Yuan K., Qin M., Zou R. (2021). Phase change material-integrated latent heat storage systems for sustainable energy solutions. Energy Environ. Sci..

[20] Faraj K., Khaled M., Faraj J., Hachem F., Castelain C. (2020). Phase change material thermal energy storage systems for cooling applications in buildings: A review. Renew. Sustain. Energy Rev..

[21] Pielichowska K., Pielichowski K. (2014). Phase change materials for thermal energy storage. Prog. Mater. Sci..

[22] Imran Khan M., Asfand F., Al-Ghamdi S.G. (2023). Progress in research and development of phase change materials for thermal energy storage in concentrated solar power. Appl. Therm. Eng..

[23] Bharathiraja R., Ramkumar T., Selvakumar M. (2023). Studies on the thermal characteristics of nano-enhanced paraffin wax phase change material (PCM) for thermal storage applications. J. Energy Storage.

[24] Peng H., Zhang D., Ling X., Li Y., Wang Y., Yu Q., She X., Li Y., Ding Y. (2018). n-Alkanes Phase Change Materials and Their Microencapsulation for Thermal Energy Storage: A Critical Review. Energy Fuels.

[25] Cui H., Wang P., Yang H., Xu T. (2022). Design and preparation of salt hydrate/graphene oxide@SiO2/SiC composites for efficient solar thermal utilization. Sol. Energy Mater. Sol. Cells.

[26] Hassan N., Minakshi M., Liew W.Y.H., Amri A., Jiang Z.-T. (2023). Thermal Characterization of Binary Calcium-Lithium Chloride Salts for Thermal Energy Storage at High Temperature. Energies.

[27] Hassan N., Minakshi M., Ruprecht J., Liew W.Y.H., Jiang Z.-T. (2023). A Binary Salt Mixture LiCl–LiOH for Thermal Energy Storage. Materials.

[28] Mehrali M., ten Elshof J.E., Shahi M., Mahmoudi A. (2021). Simultaneous solar-thermal energy harvesting and storage via shape stabilized salt hydrate phase change material. Chem. Eng. J..

[29] Cárdenas-Ramírez C., Jaramillo F., Gómez M. (2020). Systematic review of encapsulation and shape-stabilization of phase change materials. J. Energy Storage.

[30] Umair M.M., Zhang Y., Iqbal K., Zhang S., Tang B. (2019). Novel strategies and supporting materials applied to shape-stabilize organic phase change materials for thermal energy storage—A review. Appl. Energy.

[31] Chinnasamy V., Heo J., Jung S., Lee H., Cho H. (2023). Shape stabilized phase change materials based on different support structures for thermal energy storage applications—A review. Energy.

[32] Jamekhorshid A., Sadrameli S.M., Farid M. (2014). A review of microencapsulation methods of phase change materials (PCMs) as a thermal energy storage (TES) medium. Renew. Sustain. Energy Rev..

[33] Zhang S., Feng D., Shi L., Wang L., Jin Y., Tian L., Li Z., Wang G., Zhao L., Yan Y. (2021). A review of phase change heat transfer in shape-stabilized phase change materials (ss-PCMs) based on porous supports for thermal energy storage. Renew. Sustain. Energy Rev..

[34] Gandhi M., Kumar A., Elangovan R., Meena C.S., Kulkarni K.S., Kumar A., Bhanot G., Kapoor N.R. (2020). A Review on Shape-Stabilized Phase Change Materials for Latent Energy Storage in Buildings. Sustainability.

[35] Qiu L., Yan K., Feng Y., Liu X. (2023). Nano additives-enhanced PEG/AlN composites with high cycle stability to improve thermal and heat storage properties. Energy.

[36] Yan K., Feng Y., Qiu L. (2024). Thermal and photo/electro-thermal conversion characteristics of high energy storage density expanded graphite/polyethylene glycol shaped composite phase change materials. Sol. Energy.

[37] Sun S., Yan Q., Wu M., Zhao X. (2021). Carbon aerogel based materials for secondary batteries. Sustain. Mater. Technol..

[38] Lobach A.S., Kazakov V.A., Spitsyna N.G., Baskakov S.A., Dremova N.N., Shul’ga Y.M. (2017). Comparative study of graphene aerogels synthesized using sol-gel method by reducing graphene oxide suspension. High Energy Chem..

[39] Feng J., Su B.-L., Xia H., Zhao S., Gao C., Wang L., Ogbeide O., Feng J., Hasan T. (2021). Printed aerogels: Chemistry, processing, and applications. Chem. Soc. Rev..

[40] Wang M., Zhang C., Wang J., Wang Y., Yu F. (2022). Carbon hybrid aerogel-based phase change material with reinforced energy storage and electro-thermal conversion performance for battery thermal management. J. Energy Storage.

[41] Su H., Lin P., Li D., Chen Y. (2024). Reduced Graphene Oxide/Cellulose Sodium Aerogel-Supported Eutectic Phase Change Material Gel Demonstrating Superior Energy Conversion and Storage Capacity toward High-Performance Personal Thermal Management. ACS Appl. Mater. Interfaces.

[42] Deng J., Kou Y., Liu H., Yang M., Sun K., Joshi R., Shi Q. (2023). Melamine Foam/CNT/Graphene Hybrid Aerogel-Based Phase Change Composites with High Latent Heat Capacity for Solar/Electrothermal Conversion. ACS Appl. Energy Mater..

[43] Zhao J., Zhou J., Li H., Xiao A. (2023). Ti3C2Tx MXene and cellulose-based aerogel phase change composite decorated laminated fabric with excellent electro/solar-thermal conversion and high latent heat. Carbohydr. Polym..

[44] Tao Z., Zou H., Li M., Ren S., Xu J., Lin J., Yang M., Feng Y., Wang G. (2023). Polypyrrole coated carbon nanotube aerogel composite phase change materials with enhanced thermal conductivity, high solar-/electro- thermal energy conversion and storage. J. Colloid Interface Sci..

[45] He M., Lu J., Shi C., Qian X., Yin L., Gong K., Zhou K. (2023). Multi-Stimuli-Responsive Aerogels Composed of Ag Nanoparticle-Coated Graphene Nanosheets for Energy Storage and Conversion. ACS Appl. Nano Mater..

[46] Lin F., Liu X., Leng G., Bai Y., Feng J., Guo Z., Wang Z., Huang Z., Mi R., Min X. (2023). Grid structure phase change composites with effective solar/electro-thermal conversion for multi-functional thermal application. Carbon.

[47] Zhou Y., Wang X., Liu X., Sheng D., Ji F., Dong L., Xu S., Wu H., Yang Y. (2019). Polyurethane-based solid-solid phase change materials with halloysite nanotubes-hybrid graphene aerogels for efficient light- and electro-thermal conversion and storage. Carbon.

[48] Luo W., Luo L., Ma Y., Liu Y., Xie Y., Hu X., Chen W., Jiang X. (2024). Highly thermal conductive phase change materials enabled by CNTs-modified PVA aerogel for solar energy storage and thermal management of electronic components. J. Energy Storage.

[49] Ai H., Lv L., Chen T., Zhang Y., Dong L., Song S. (2022). An eco-friendly and facile montmorillonite nanosheets aerogel based phase change materials for efficient solar-to-thermal energy conversion. Energy Convers. Manag..

[50] Li Y., Hu G., Wang Q., Dong F., Xiong Y. (2024). Design of PVA/multilayer hybrid particle composite aerogel skeleton supported form-stable phase change materials with high thermal conductivity and solar-to-thermal conversion efficiency. J. Energy Storage.

[51] Zhu L., Zou B., Bing N., Xie H., Yu W. (2024). Bidirectional anisotropic bacterial cellulose/polyvinyl alcohol/MXene aerogel phase change composites for photothermal conversion enhancement. Sol. Energy Mater. Sol. Cells.

[52] (2022). In situ preparation of light-driven cellulose-Mxene aerogels based composite phase change materials with simultaneously enhanced light-to-heat conversion, heat transfer and heat storage. Compos. Part A Appl. Sci. Manuf..

[53] Liu Y., Liu H., Qi H. (2023). High efficiency electro- and photo-thermal conversion cellulose nanofiber-based phase change materials for thermal management. J. Colloid Interface Sci..

[54] Sun Z., Zhang H., Zhang Q., Jing R., Wu B., Xu F., Sun L., Xia Y., Rosei F., Peng H. (2022). Shape-stabilized phase change composites enabled by lightweight and bio-inspired interconnecting carbon aerogels for efficient energy storage and photo-thermal conversion. J. Mater. Chem. A.

[55] Gui H., Zhao X., Zuo S., Liu W., Wang C., Xu P., Ding Y., Yao C. (2023). Carbonized Syndiotactic Polystyrene/Carbon Nanotube/MXene Hybrid Aerogels with Egg-Box Structure: A Platform for Electromagnetic Interference Shielding and Solar Thermal Energy Management. ACS Appl. Mater. Interfaces J..

[56] Tang Y., Cheng Z., Yue H., Wang X., Wang H., Du Z., Cheng X., Dai R., Du X., Wu D. (2024). Nb2CTx MXene/Delignified Wood–Supported Phase-Change Composites with Desirable Photothermal Conversion Efficiency and Enhanced Flame Retardancy for Solar–Thermal Energy Storage. ACS Appl. Energy Mater..

[57] Shu C., Zhao H.-Y., Lu X.-H., Min P., Zhang Y., Wang Q., Li X., Yu Z.-Z. (2023). High-Quality Anisotropic Graphene Aerogels and Their Thermally Conductive Phase Change Composites for Efficient Solar–Thermal–Electrical Energy Conversion. ACS Sustain. Chem. Eng..

[58] Lin W., Lai J., Xie K., Liu D., Wu K., Fu Q. (2022). D-Mannitol/Graphene Phase-Change Composites with Structured Conformation and Thermal Pathways Allow Durable Solar–Thermal–Electric Conversion and Electricity Output. ACS Appl. Mater. Interfaces.

[59] Han S., Xiong F., Qin M., Shen Z., Han H., Jin Y., Usman A., Wang Y., Zhong R., Zou R. (2024). Polyethylene glycol/polypyrrole aerogel shape-stabilized phase change material for solar-thermal energy storage and thermoelectric power generation. Sol. Energy Mater. Sol. Cells.

[60] Du X., Wang J., Jin L., Deng S., Dong Y., Lin S. (2022). Dopamine-Decorated Ti_3_C_2_T_x_ MXene/Cellulose Nanofiber Aerogels Supported Form-Stable Phase Change Composites with Superior Solar–Thermal Conversion Efficiency and Extremely High Thermal Storage Density. ACS Appl. Mater. Interfaces.

[61] Guo Q., Yi H., Jia F., Song S. (2024). Novel MoS2/montmorillonite hybrid aerogel encapsulated PEG as composite phase change materials with superior solar-thermal energy harvesting and storage. J. Colloid Interface Sci..

[62] Song S., Ai H., Zhu W., Lv L., Feng R., Dong L. (2021). Carbon aerogel based composite phase change material derived from kapok fiber: Exceptional microwave absorbility and efficient solar/magnetic to thermal energy storage performance. Compos. Part B Eng..

[63] Shen R., Weng M., Zhang L., Huang J., Sheng X. (2022). Biomass-based carbon aerogel/Fe_3_O_4_@PEG phase change composites with satisfactory electromagnetic interference shielding and multi-source driven thermal management in thermal energy storage. Compos. Part A Appl. Sci. Manuf..

[64] Jin L., Han Q., Wang J., Wang S., Wang H., Cheng X., Du X., Du Z. (2023). Fe_3_O_4_-Functionalized κ-Carrageenan/Melanin Hybrid Aerogel-Supported Form-Stable Phase-Change Composites with Excellent Solar/Magnetic–Thermal Conversion Efficiency and Enhanced Thermal Conductivity. ACS Sustain. Chem. Eng..

[65] Tao Z., Yang M., Wu L., Yan J., Yang F., Lin J., Wang J., Wang G. (2021). Phase change material based on polypyrrole/Fe_3_O_4_-functionalized hollow kapok fiber aerogel matrix for solar/magnetic-thermal energy conversion and storage. Chem. Eng. J..

[66] Hu B., Guo H., Li T., Cao X., Cao M., Qi W., Cui Y., Li B. (2025). Engineering tiramisu-like phase change nanocomposite for superior thermal energy management and electromagnetic interference shielding. J. Mater. Sci. Technol..

[67] Liu L., Hu J., Fan X., Zhang Y., Zhang S., Tang B. (2021). Phase change materials with Fe_3_O_4_/GO three-dimensional network structure for acoustic-thermal energy conversion and management. Chem. Eng. J..

[68] Liu M., Qian R., Yang Y., Lu X., Huang L., Zou D. (2024). Modification of Phase Change Materials for Electric-Thermal, Photo-Thermal, and Magnetic-Thermal Conversions: A Comprehensive Review. Adv. Funct. Mater..

[69] (2022). High latent heat phase change materials (PCMs) with low melting temperature for thermal management and storage of electronic devices and power batteries: Critical review. Renew. Sustain. Energy Rev..

[70] Zhang Y., Umair M.M., Zhang S., Tang B. (2019). Phase change materials for electron-triggered energy conversion and storage: A review. J. Mater. Chem. A.

[71] Jia Z., Hu C., Zhang Y., Zhang S., Tang B. (2023). Exploring electro-thermal conversion in phase change materials: A review. Compos. Part A Appl. Sci. Manuf..

[72] Shchukina E.M., Graham M., Zheng Z., Shchukin D.G. (2018). Nanoencapsulation of phase change materials for advanced thermal energy storage systems. Chem. Soc. Rev..

[73] Okuda H., Young R.J., Wolverson D., Tanaka F., Yamamoto G., Okabe T. (2018). Investigating nanostructures in carbon fibres using Raman spectroscopy. Carbon.

[74] Chen X., Tang Z., Gao H., Chen S., Wang G. (2020). Phase Change Materials for Electro-Thermal Conversion and Storage: From Fundamental Understanding to Engineering Design. iScience.

[75] Li Y., Samad Y.A., Polychronopoulou K., Alhassan S.M., Liao K. (2014). From biomass to high performance solar–thermal and electric–thermal energy conversion and storage materials. J. Mater. Chem. A.

[76] Pandey A.K., Hossain M.S., Tyagi V.V., Abd Rahim N., Jeyraj A., Selvaraj L., Sari A. (2018). Novel approaches and recent developments on potential applications of phase change materials in solar energy. Renew. Sustain. Energy Rev..

[77] Qiu L., Ouyang Y., Feng Y., Zhang X. (2019). Review on micro/nano phase change materials for solar thermal applications. Renew. Energy.

[78] Shi L., Wang X., Hu Y., He Y., Yan Y. (2020). Solar-thermal conversion and steam generation: A review. Appl. Therm. Eng..

[79] Yeshchenko O.A., Kutsevol N.V., Naumenko A.P. (2016). Light-Induced Heating of Gold Nanoparticles in Colloidal Solution: Dependence on Detuning from Surface Plasmon Resonance. Plasmonics.

[80] Ibrahim I., Seo D.H., McDonagh A.M., Shon H.K., Tijing L. (2021). Semiconductor photothermal materials enabling efficient solar steam generation toward desalination and wastewater treatment. Desalination.

[81] Miao E.-D., Ye M.-Q., Guo C.-L., Liang L., Liu Q., Rao Z.-H. (2019). Enhanced solar steam generation using carbon nanotube membrane distillation device with heat localization. Appl. Therm. Eng..

[82] Li G., Wang Y., Zhang X. (2021). Graphene aerogel-phase change material host-guest smart films. FlatChem.

[83] Yang H., Chao W., Di X., Yang Z., Yang T., Yu Q., Liu F., Li J., Li G., Wang C. (2019). Multifunctional wood based composite phase change materials for magnetic-thermal and solar-thermal energy conversion and storage. Energy Convers. Manag..

[84] (2005). Investigation of convective heat transfer augmentation using acoustic streaming generated by ultrasonic vibrations. Int. J. Heat Mass Transf..

[85] Cui W., Li X., Li X., Lu L., Ma T., Wang Q. (2022). Combined effects of nanoparticles and ultrasonic field on thermal energy storage performance of phase change materials with metal foam. Appl. Energy.

[86] Wang G., Tang Z., Gao Y., Liu P., Li Y., Li A., Chen X. (2023). Phase Change Thermal Storage Materials for Interdisciplinary Applications. Chem. Rev..

[87] Wan L., Liu C., Cao D., Sun X., Zhu H. (2020). High Phase Change Enthalpy Enabled by Nanocellulose Enhanced Shape Stable Boron Nitride Aerogel. ACS Appl. Polym. Mater..

[88] Wei X., Xue F., Qi X., Yang J., Zhou Z., Yuan Y., Wang Y. (2019). Photo- and electro-responsive phase change materials based on highly anisotropic microcrystalline cellulose/graphene nanoplatelet structure. Appl. Energy.

[89] Zhong Y., Zhou M., Huang F., Lin T., Wan D. (2013). Effect of graphene aerogel on thermal behavior of phase change materials for thermal management. Sol. Energy Mater. Sol. Cells.

[90] Cheng M., Hu J., Xia J., Liu Q., Wei T., Ling Y., Li W., Liu B. (2022). One-step in-situ green synthesis of cellulose nanocrystal aerogel based shape stable phase change material. Chem. Eng. J..

[91] Tian B., Yang W., He F., Xie C., Zhang K., Fan J., Wu J. (2017). Paraffin/carbon aerogel phase change materials with high enthalpy and thermal conductivity. Fuller. Nanotub. Carbon Nanostruct..

[92] Zhang L., An L., Wang Y., Lee A., Schuman Y., Ural A., Fleischer A.S., Feng G. (2019). Thermal enhancement and shape stabilization of a phase-change energy-storage material via copper nanowire aerogel. Chem. Eng. J..

[93] Chen X., Cheng P., Tang Z., Xu X., Gao H., Wang G. (2021). Carbon-Based Composite Phase Change Materials for Thermal Energy Storage, Transfer, and Conversion. Adv. Sci..

[94] Wang B., Li G., Xu L., Liao J., Zhang X. (2020). Nanoporous Boron Nitride Aerogel Film and Its Smart Composite with Phase Change Materials. ACS Nano.

[95] Shi J., Qin M., Aftab W., Zou R. (2021). Flexible phase change materials for thermal energy storage. Energy Storage Mater..

[96] Zhao T., Zhang T., Xu Z., Zhao Y. (2022). Emulsion-based, flexible and recyclable aerogel composites for latent heat storage. J. Colloid Interface Sci..

[97] Cai Y., Zhang N., Cao X., Yuan Y., Zhang Z., Yu N. (2023). Ultra-light and flexible graphene aerogel-based form-stable phase change materials for energy conversion and energy storage. Sol. Energy Mater. Sol. Cells.

[98] Lyu J., Li G., Liu M., Zhang X. (2019). Aerogel-Directed Energy-Storage Films with Thermally Stimulant Multiresponsiveness. Langmuir.

[99] Pang H.-Q., Zhang R., Yang H.-L., Li Z.-Y., Xu H.-B. (2022). Preparation and thermal insulation performance characterization of endothermic opacifier doped silica aerogel. Int. J. Therm. Sci..

[100] Zhang M., Xiao Q., Chen C., Li L., Yuan W. (2020). Developing a heat-insulating composite phase change material with light-to-thermal conversion performance from graphene oxide/silica hybrid aerogel. Appl. Therm. Eng..

[101] Ding J., Wu X., Shen X., Cui S., Chen X. (2020). A promising form-stable phase change material composed of C/SiO_2_ aerogel and palmitic acid with large latent heat as short-term thermal insulation. Energy.

[102] Liu Z., Lyu J., Fang D., Zhang X. (2019). Nanofibrous Kevlar Aerogel Threads for Thermal Insulation in Harsh Environments. ACS Nano.

[103] Liu P., Gao H., Chen X., Chen D., Lv J., Han M., Cheng P., Wang G. (2020). In situ one-step construction of monolithic silica aerogel-based composite phase change materials for thermal protection. Compos. Part B Eng..

[104] Xiangfa Z., Hanning X., Jian F., Changrui Z., Yonggang J. (2012). Preparation, properties and thermal control applications of silica aerogel infiltrated with solid–liquid phase change materials. J. Exp. Nanosci..

[105] Pielichowska K., Paprota N., Pielichowski K. (2023). Fire Retardant Phase Change Materials—Recent Developments and Future Perspectives. Materials.

[106] Du X., Qiu J., Deng S., Du Z., Cheng X., Wang H. (2020). Flame-retardant and form-stable phase change composites based on black phosphorus nanosheets/cellulose nanofiber aerogels with extremely high energy storage density and superior solar-thermal conversion efficiency. J. Mater. Chem. A.

[107] Luo Y., Xie Y., Jiang H., Chen Y., Zhang L., Sheng X., Xie D., Wu H., Mei Y. (2021). Flame-retardant and form-stable phase change composites based on MXene with high thermostability and thermal conductivity for thermal energy storage. Chem. Eng. J..

[108] Li J., Chen R., Luo Y., Shi J., Sheng X., Xie Y., Wu H., Xie D., Mei Y. (2022). SWCNT-Encapsulated Phosphorus-Grafted Stearyl Alcohol as a Flame-Retardant Phase-Change Material with Superior Thermal Properties. ACS Appl. Energy Mater..

[109] Zhou M., Xie D., Zhou K., Gong K., Yin L., Qian X., Shi C. (2022). 3D porous aerogel based-phase change materials with excellent flame retardancy and shape stability for both thermal and light energy storage. Sol. Energy Mater. Sol. Cells.

[110] Cao F., Li Z., Zhang Y., Wang X., Zhu L., Zhang S., Tang B. (2024). Silica-based aerogels encapsulate organic/inorganic composite phase change materials for building thermal management. J. Energy Storage.

[111] Cao Y., Weng M., Mahmoud M.H.H., Elnaggar A.Y., Zhang L., El Azab I.H., Chen Y., Huang M., Huang J., Sheng X. (2022). Flame-retardant and leakage-proof phase change composites based on MXene/polyimide aerogels toward solar thermal energy harvesting. Adv. Compos. Hybrid Mater..

